# nPCA: a linear dimensionality reduction method using a multilayer perceptron

**DOI:** 10.3389/fgene.2023.1290447

**Published:** 2024-01-08

**Authors:** Juzeng Li, Yi Wang

**Affiliations:** ^1^ Ministry of Education Key Laboratory of Contemporary Anthropology, Department of Anthropology and Human Genetics, School of Life Sciences, Fudan University, Shanghai, China; ^2^ Human Phenome Institute, Fudan University, Shanghai, China

**Keywords:** linear dimensionality reduction, neural principal component analysis, single-cell RNA sequencing, multilayer perceptron, activation function

## Abstract

**Background:** Linear dimensionality reduction techniques are widely used in many applications. The goal of dimensionality reduction is to eliminate the noise of data and extract the main features of data. Several dimension reduction methods have been developed, such as linear-based principal component analysis (PCA), nonlinear-based t-distributed stochastic neighbor embedding (t-SNE), and deep-learning-based autoencoder (AE). However, PCA only determines the projection direction with the highest variance, t-SNE is sometimes only suitable for visualization, and AE and nonlinear methods discard the linear projection.

**Results:** To retain the linear projection of raw data and generate a better result of dimension reduction either for visualization or downstream analysis, we present neural principal component analysis (nPCA), an unsupervised deep learning approach capable of retaining richer information of raw data as a promising improvement to PCA. To evaluate the performance of the nPCA algorithm, we compare the performance of 10 public datasets and 6 single-cell RNA sequencing (scRNA-seq) datasets of the pancreas, benchmarking our method with other classic linear dimensionality reduction methods.

**Conclusion:** We concluded that the nPCA method is a competitive alternative method for dimensionality reduction tasks.

## 1 Introduction

The dimensionality reduction method produces a low-dimensional linear mapping of the original high-dimensional data, and it can be used for visualizing data, denoising or compressing scRNA-seq data, and extracting meaningful feature spaces (Zebari et al., 2020). The dimensionality reduction methods are commonly divided into linear and nonlinear approaches (Van Der Maaten et al., 2009). Several classical and representative linear dimensionality reduction methods are widely used in biological data analysis, including independent component analysis (ICA) (Stone, 2002), multidimensional scaling (MDS) (Hout et al., 2013), factor analysis (FA), and principal component analysis (PCA) (Wold et al., 1987). In addition, nonlinear methods, such as t-distributed stochastic neighbor embedding (t-SNE) (Van der Maaten and Hinton, 2008) and uniform manifold approximation and projection (UMAP) (McInnes et al., 2018), are also widely used in processing biological big data. Each method has its own features and limitations.

PCA is a widely used linear dimensionality reduction algorithm (Wold et al., 1987) that calculates the first principal component with the largest variance and then seeks the second component in the same manner, which is uncorrelated with the first component and accounts for the next largest variance (Xiang et al., 2021). Furthermore, the autoencoder (AE) is a nonlinear generalization of PCA that uses a multilayer encoder network to transform the high-dimensional data into a low-dimensional code and a similar decoder network to recover the data from the code (Hinton and Salakhutdinov, 2006). Therefore, in order to combine the advantages of a linear encoder and nonlinear decoder, we developed the neural principal component analysis (nPCA) method, which is a linear dimensionality reduction algorithm using the deep learning method (multilayer perceptron). We also list a comparison of encoding and decoding modalities between different methods (Table 1). The nPCA algorithm is more like a transition between linear and nonlinear methods. It uses the nonlinear decoder approach but holds the linear encoder (linear projection of raw data). Furthermore, when the dimensionality reduction results are produced after training, the nonlinear decoder will be discarded. In other words, nPCA is another linear dimensionality reduction method that is an upgrade to PCA.

**TABLE 1:** Modalities of the encoder and decoder in three methods.

Method	Encoder	Decoder
PCA	Linear	Linear
nPCA	Linear	Nonlinear
Autoencoder	Nonlinear	Nonlinear

Considering that PCA determines the projection direction with the largest variance, it is not the projection direction that retains the most information from the original data. In order to solve this problem, nPCA uses the deep learning method to gradually correct the projection matrix to achieve this goal. In this paper, we compared the performance of nPCA with that of PCA, using 10 publicly available datasets (mostly related to biology), to verify that nPCA holds richer information of raw data than PCA. Then, the performance of nPCA and three other linear dimensionality reduction methods were tested on six single-cell RNA-seq datasets of the pancreas.

## 2 Materials and methods

### 2.1 Methodological framework of nPCA

**FIGURE 1:**
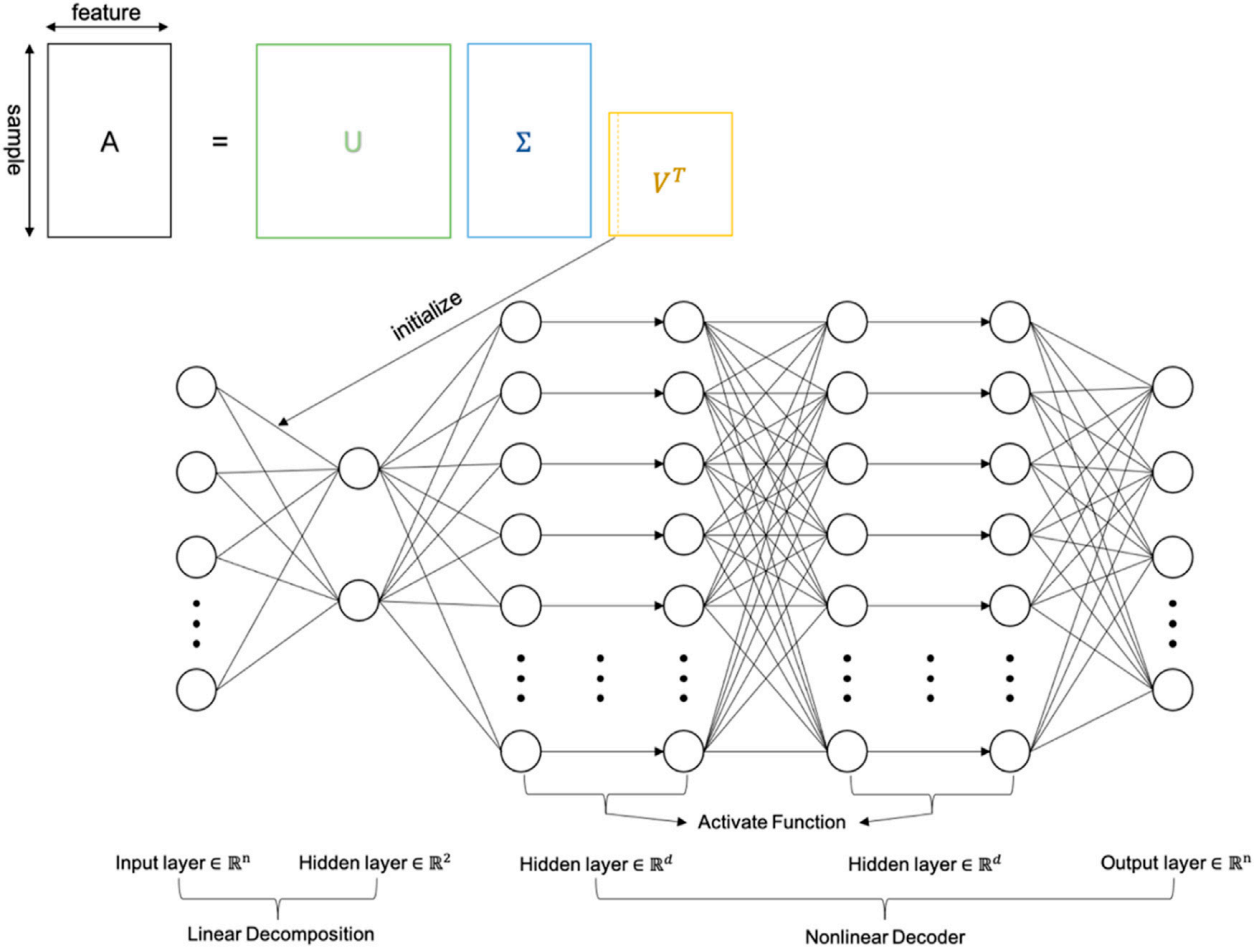
Structure of nPCA.

We propose an nPCA approach to hold richer structural information of the raw data than PCA. The proposed approach is based on a multilayer perceptron (MLP), which is a type of artificial neural network (ANN) (Mac et al., 2022). The nPCA we designed comprises an input layer, three hidden layers, and an output layer. The number of neurons in the input and output layers is the same as the dimension of the original data, and the first hidden layer has two neurons, and the remaining two hidden layers have d neurons each (we use 32 neurons for nPCA). The activation function plays an important role in ANNs because it is directly linked with obtained success rates (Ertugrul, 2018). However, in nPCA, we remove the activation function of the first hidden layer to ensure that the neurons of the first layer are only the linear projection of the original data, and the following layers act as a nonlinear decoder for these two neurons (Figure 1). The definition of the loss function comprising the output layer ( y ) and input layer ( x ) is as follows:

$$E=\frac{1}{2}\sum_{i}^{n}{\left(y_{i}-x_{i}\right)}^{2}.$$

We then use the stochastic gradient descent (SGD) (Bottou, 2012) to minimize E in backpropagation. When the value of E decreases to the minimum, the training is terminated.

The two neurons of the first hidden layer are the two principal components generated by nPCA. Similar to the traditional linear dimensionality reduction method, a linear transformation is carried out on the raw data, but due to the existence of the nonlinear decoder, nPCA will retain more information on the original data.

Given an n×m data matrix A with n samples and m features, we first perform singular value decomposition (SVD) on this matrix A , using the two eigenvectors of matrix U to initialize the weights between the input layer and the first hidden layer (Figure 1). The selected eigenvectors correspond to the two largest eigenvalues in matrix Σ , which is the dimensionality reduction method of PCA. Other weights are randomly initialized.

### 2.2 Benchmarking

We benchmarked five linear dimensionality reduction methods: ICA, MDS, FA, PCA and nPCA. Our nPCA and PCA methods are implemented using our own C++ code, and these two methods are used in public datasets. The other two methods are implemented using a Python package sklearn. In addition, the comparison of the four methods is performed in single-cell datasets. Since our original intention of designing nPCA is to upgrade PCA, we use the same multilayer perceptron structure for PCA training in order to confirm that nPCA retains more information than PCA. However, the difference is that in the PCA network, we always fix the weight of the first layer to be the linear dimensionality reduction parameter of PCA, that is, the weight of the first layer is not updated when SGD is used every time. This facilitates to achieve the result that after training if the loss of nPCA is less than that of PCA, it proves that nPCA retains more raw data information than PCA. Benchmarking was performed on a desktop PC equipped with an Intel Core i7-11700 CPU and 32 GB of memory.

### 2.3 Benchmarking public dataset study

Overall, 10 public datasets were included: housing (Belsley et al., 2005), bike sharing (Fanaee-T and Gama, 2014), Anuran calls (Colonna et al., 2015), telemonitoring of Parkinson’s disease (Tsanas et al., 2009), QSAR biodegradation (Mansouri et al., 2013), Indian liver patient (Ramana et al., 2012), blood transfusion service center (Yeh et al., 2009), iris (Fisher, 1936), seed (Charytanowicz et al., 2010), and cervical cancer behavior risk (Sobar and Wijaya, 2016).

### 2.4 Application to scRNA-seq dataset study

To evaluate the performance of nPCA, we applied each of the aforementioned methods to the six single-cell gene expression datasets of the pancreas (Baron et al., 2016), which included four human and two mouse datasets.

Before using the scRNA-seq data, a Python package Scanpy was used to preprocess the data and quality control. The first step in preprocessing involves filtering out the weakly expressed genes and low-quality cells and removing those genes expressed in less than three cells and cells that expressed less than 200 genes (Vasighizaker et al., 2022). Second, because mitochondrial genes do not carry important information required for downstream analysis (Ilicic et al., 2016), those cell samples with mitochondrial genes are removed, accounting for more than 95%. Furthermore, in order to remove the influence of potential variation, we screen out those cell samples with more than 2,500 expressed genes (Vasighizaker et al., 2022). Third, since scRNA-seq data are expressed at a different level, we normalize the data using the following formula:

$$readsMappedToGene\times \frac{1}{totalReads}\times 10^{4}$$

and use the logarithmic transformation on the normalized data:

$$f\left(x\right)=\log_{10}\left(x+1\right).$$

In the end, the three subsets of 200, 500, and 1,000 features were extracted in one dataset by highly variable genes.

We performed four linear dimensionality reduction methods, namely, ICA, MDS, PCA, and nPCA, to reduce the original data ( n×m matrix) to two-dimensional data ( n×2 matrix). First, in the case of not providing the real label but providing the number of categories, we apply the popular clustering technique, k-means, to the data after dimensionality reduction and calculate the mean silhouette coefficient (SH) (Řezanková, 2018) of all samples to evaluate the quality of the clustering effect. SH for one sample can be defined as follows:

$$SH\left(x_{i}\right)=\frac{\left[b\left(x_{i}\right)\;-\;a\left(x_{i}\right)\right]}{\max\;\left[a\left(x_{i}\right),b\left(x_{i}\right)\right]},$$

where $a\left(x_{i}\right)$ is the intra-cluster dissimilarity: the average value of dissimilarity between the sample $x_{i}$ vector and other samples in the same cluster; $b\left(x_{i}\right)$ is the inter-cluster dissimilarity: the minimum value of the average dissimilarity between the sample $x_{i}$ vector and other clusters. Then, the true label is provided and the adjusted Rand index (ARI) (Sundqvist et al., 2022) is calculated to compare the performance of four linear dimensionality reduction methods. Given two clustering groups X and Y , the following four quantities are defined:

a : the number of objects in a pair placed in the same group in X and in the same group in Y .

b : the number of objects in a pair placed in the different group in X and in the different group in Y .

c : the number of objects in a pair placed in the same group in X and in the different group in Y .

d : the number of objects in a pair placed in the different group in X and in the same group in Y .

Then, ARI is proposed in the form of

$$ARI=\frac{\left(\begin{array}{l} n\\ 2 \end{array}\right)\left(a+b\right)-\;\left[\left(a+b\right)\left(a+c\right)+\left(c+d\right)\left(b+d\right)\right]}{\left(\begin{array}{l} n\\ 2 \end{array}\right)-\left[\left(a+b\right)\left(a+c\right)+\left(c+d\right)\left(b+d\right)\right]}.$$

At last, the dimensionality-reduced data were further visualized using t-SNE to compare the four methods.

## 3 Result

### 3.1 Results from public datasets

Table 2 shows the loss values at the completion of training for PCA and nPCA on 10 public datasets. nPCA performs better in 8 of the 10 datasets. We calculate the variance captured using the following formula:

$$Vaeiance\;captured=\left(1-2\times E\right)\times 100\%.$$

**TABLE 2:** Variance captured (loss) of PCA and nPCA on 10 datasets. Values in bold indicate the first-place result of the two methods compared.

Dataset	Sample	Feature	Variance captured of PCA (%)	Variance captured of nPCA (%)
Housing	506	14	79.63	**87.30**
Bike	17,379	16	42.52	**57.00**
Anuran calls (MFCCs)	7,195	22	62.05	**70.78**
Telemonitoring of Parkinson’s disease	5,875	21	71.92	**73.11**
QSAR biodegradation	1,055	41	48.89	**54.82**
Indian liver patient	582	10	92.43	**95.87**
Blood transfusion service center	748	4	**97.81**	97.48
Iris	150	4	99.75	**99.82**
Seeds	210	7	**98.64**	98.19
Cervical cancer behavior risk	72	19	96.90	**97.26**

Here, E is the loss value after training. Because we use the L2 loss function, if PCA and nPCA dimension reduction results contain more information of the original data, the lower loss value (higher variance captured) is obtained after it propagates through the same network. So this result has proven that nPCA can retain more information of the original data than PCA.

**FIGURE 2:**
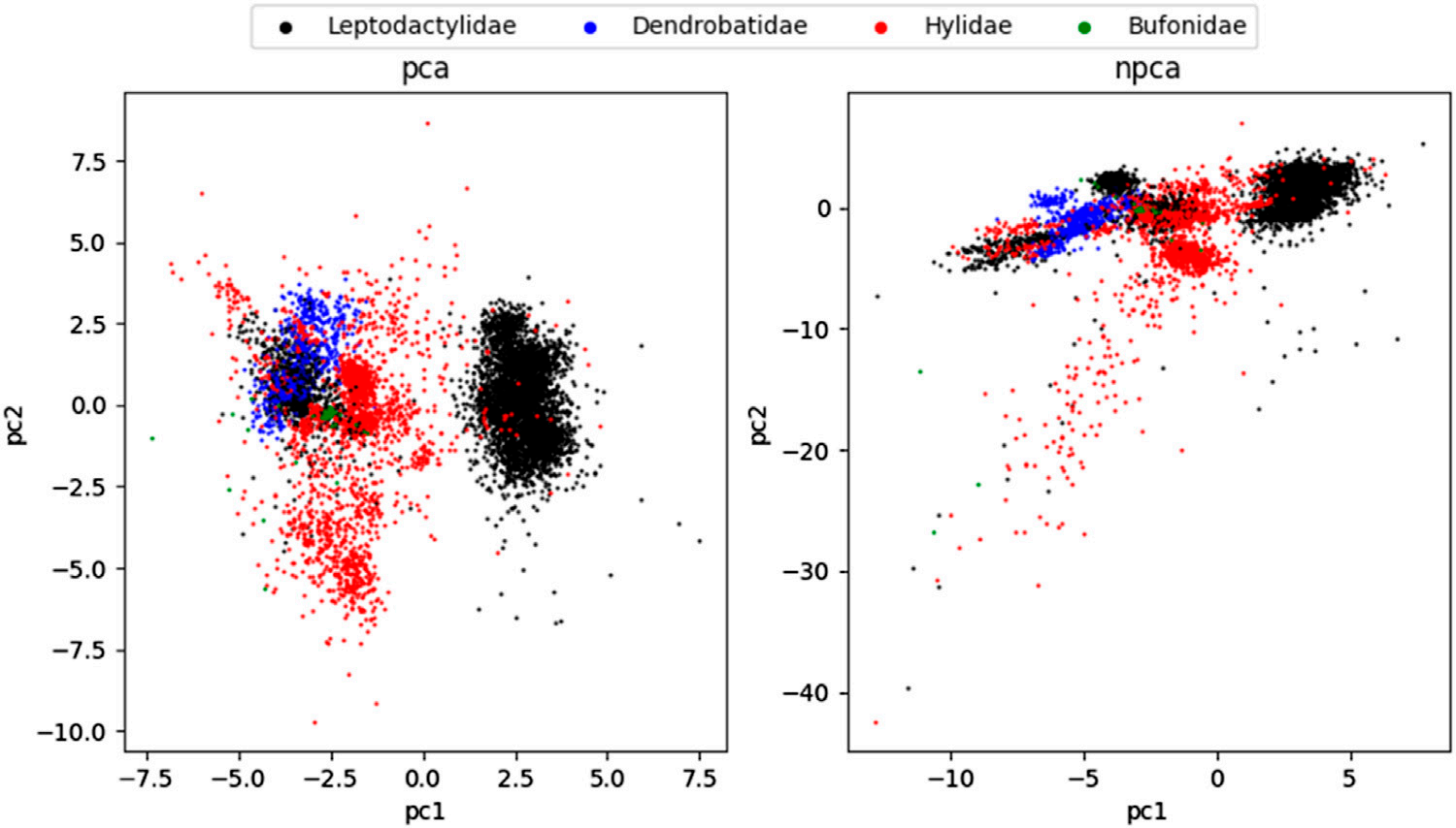
Two-dimensional PCA and nPCA projection of the Anuran calls (MFCCs) dataset colored by a real label (family of Anuran).

Figure 2 shows the results of PCA and nPCA of the Anuran calls (MFCCs) dataset. We can see that the Dendrobatidae family is not separated in PCA but separated from other families in nPCA. From the numerical results and figures, it is proved that nPCA retains more original data information than PCA while guaranteeing a linear dimensionality reduction method. Visualization results for the rest of the data are shown in the Supplementary File.

Since there is a training process in nPCA, we also saved the results of the training process of the Anuran calls (MFCCs) dataset to generate 90 pictures of the process and made a GIF animation (Supplementary File). Thus, nPCA is a competitive method for linear dimensionality reduction tasks.

### 3.2 Results from scRNA-seq datasets

We benchmarked four linear dimensionality reduction methods, namely, ICA, MDS, PCA, and nPCA, in the scRNA-seq dataset.

Table 3 shows the adjusted Rand score with ground truth labels and the silhouette score without ground truth labels after using four linear dimensionality reduction methods and k-means clustering (with the number of categories of real data) on 18 datasets (each dataset is divided into three sub-datasets by highly variable genes). Similarly, there is a comparison of loss between PCA and nPCA after training. This table shows that comparing the adjusted Rand score, nPCA performs best on seven datasets, MDS on five, PCA on four, and ICA only on two. Comparing the silhouette score, nPCA performs optimally on 16 datasets and PCA performs best on two datasets. Through a comprehensive comparison of internal and external clustering evaluation metrics, we observed that among all linear dimensionality reduction methods, nPCA demonstrates outstanding performance in clustering. Utilizing internal evaluation metrics (silhouette score) and external evaluation metrics (adjusted Rand score), nPCA consistently outperforms the other three linear dimensionality reduction methods. This result strongly supports the superior performance of nPCA in these tasks. In addition, the value of loss also proves that nPCA retains more raw data information than PCA on these datasets.

**TABLE 3:** Adjusted Rand score (with k-means and true label) and silhouette score (with k-means) of four linear dimensionality reduction methods on 18 datasets. Variance captured (loss) of PCA and nPCA. Significant values are represented in bold (HVG, highly variable gene).

	—	Adjusted Rand score					Silhouette score					Variance captured	
Database name	HVG	ICA	MDS	FA	PCA	nPCA	ICA	MDS	FA	PCA	nPCA	PCA	nPCA
Baron_human1	200	0.253	0.195	0.235	0.352	**0.373**	0.437	0.335	0.503	0.465	**0.773**	40.21%	**41.92%**
	500	0.258	0.208	0.255	**0.376**	0.35	0.397	0.372	0.475	0.72	**0.776**	33.46%	**40.92%**
	1,000	0.298	0.248	0.33	0.384	**0.57**	0.396	0.377	0.514	**0.67**	0.577	26.77%	**33.42%**
Baron_human2	200	0.224	**0.305**	0.188	0.218	0.168	0.593	0.36	0.68	0.477	**0.825**	45.79%	**55.37%**
	500	0.219	0.292	0.221	0.225	**0.583**	0.37	0.351	0.448	0.401	**0.61**	37.62%	**40.05%**
	1,000	0.292	**0.371**	0.249	0.255	0.246	0.359	0.352	0.445	0.423	**0.858**	31.11%	**35.93%**
Baron_human3	200	**0.208**	0.202	0.159	0.175	0.176	0.521	0.382	**0.646**	0.64	0.559	24.94%	**35.94%**
	500	0.406	0.394	0.191	0.234	**0.615**	0.402	0.352	**0.65**	0.427	0.521	20.97%	**29.31%**
	1,000	0.336	0.39	0.363	0.421	**0.663**	0.358	0.383	0.487	0.491	**0.582**	21.26%	**25.46%**
Baron_human4	200	0.205	0.156	0.151	**0.236**	0.06	0.472	0.363	0.648	0.635	**0.928**	35.93%	**56.61%**
	500	0.217	0.242	0.235	0.221	**0.612**	0.357	0.357	0.501	0.386	**0.567**	41.46%	**42.77%**
	1,000	**0.372**	0.303	0.243	0.254	0.237	0.355	0.376	0.419	0.454	**0.806**	28.42%	**31.26%**
Baron_mouse1	200	0.194	**0.371**	0.243	0.276	0.257	0.383	0.364	0.676	0.433	**0.842**	44.25%	**44.75%**
	500	0.373	0.394	0.283	0.372	**0.684**	0.457	0.372	0.543	0.491	**0.716**	29.21%	**36.61%**
	1,000	0.399	0.32	0.428	**0.523**	0.483	0.427	0.375	0.523	0.454	**0.651**	22.25%	**27.34%**
Baron_mouse2	200	0.287	0.188	0.28	**0.353**	0.194	0.479	0.318	0.732	0.465	**0.834**	50.13%	**55.80%**
	500	0.252	**0.391**	0.287	0.289	0.304	0.452	0.342	0.809	0.834	**0.878**	34.80%	**43.62%**
	1,000	0.209	**0.324**	0.294	0.298	0.235	0.435	0.361	0.826	0.839	**0.859**	31.84%	**32.22%**

**FIGURE 3:**
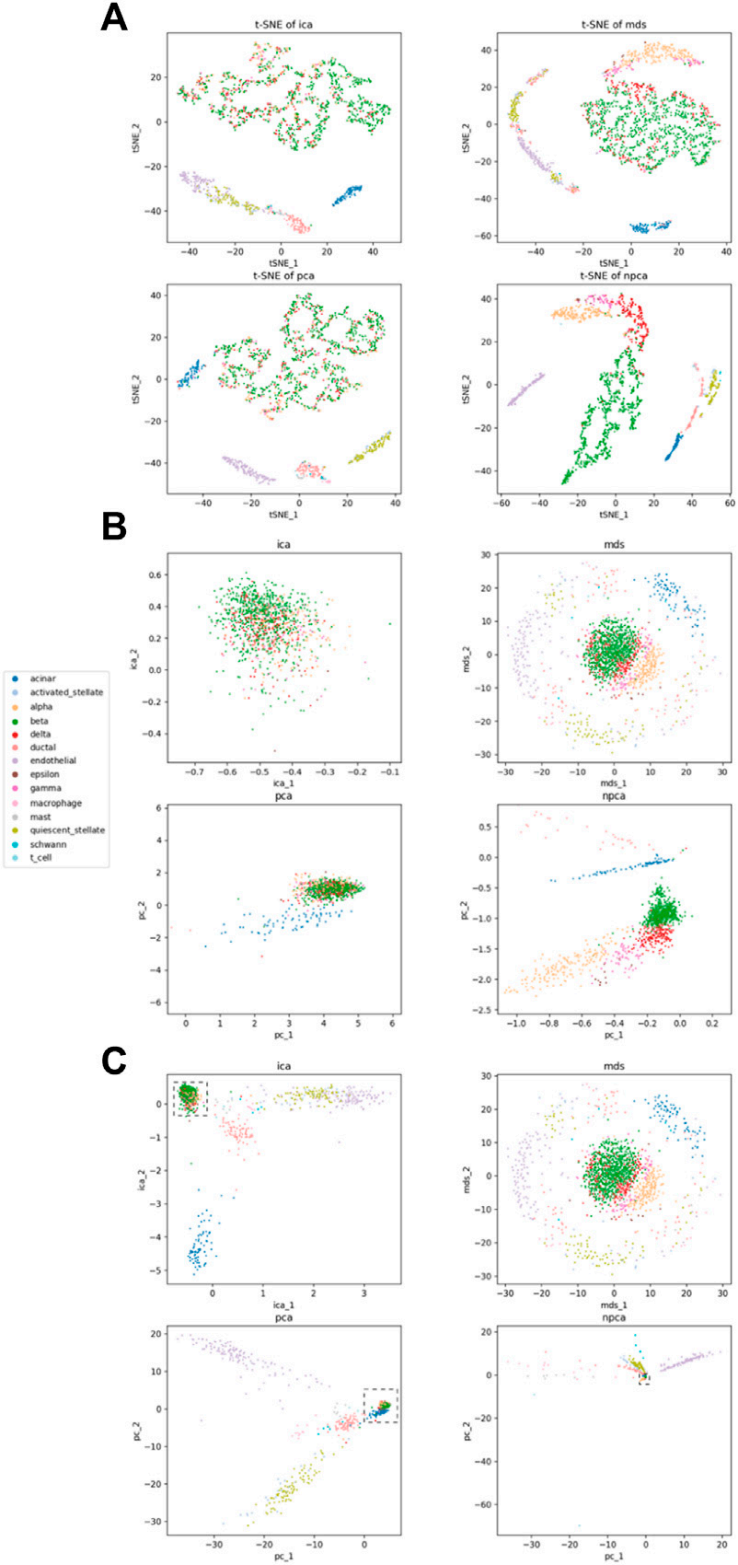
Visualization for Baron_human1 of 1,000 highly variable genes. **(A)** Comparison of four linear methods using t-SNE; **(B)** partially enlarged view of **(C)**; **(C)** comparison of four linear methods. Each point represents a cell and is colored by the real label.

In order to further visualize the results, we used t-SNE to cluster the two-dimensional result data that were outputted using four linear dimensionality reduction methods. Figure 3A shows the result of t-SNE. It can be clearly seen that the effect of dimensionality reduction of nPCA is better than the other three methods. Figure 3C shows the results of four methods for the Baron_human1 dataset of 1,000 highly variable genes. Figure 3B shows an enlarged part of the dotted box in Figure 3C. In the enlarged figure, we can see the categories that are not distinguished in the other three methods, but nPCA can achieve a better discrimination effect.

In addition, on the Baron_human1 dataset of 200 highly variable genes, no significant difference is observed in the visualization results of the four methods. On the 500 highly variable genes, nPCA gradually shows better results than the other three methods, but the effect is most obvious when there are 1,000 highly variable genes. The aforementioned results and the remaining data (in Table 3) are shown in the Supplementary File.

Overall, using the linear dimensionality reduction method on scRNA-seq requires adequate features to obtain valid results. In addition, nPCA, under the same conditions, provides better projection results than the existing linear dimensionality reduction methods. Although the evaluation index of nPCA was not suitable for all datasets, it was the top performer in both the public and scRNA-seq datasets.

## 4 Discussion

With the technological advances in scRNA-seq, the analysis methods applied to it are also constantly updated (Chen et al., 2019). However, most of the methods revolve around the aspect of clustering (e.g., t-SNE), which performs a nonlinear transformation on the data. The recent dimension reduction methods used for scRNA-seq include SinNLRR (Zheng et al., 2019), SIMLR (Wang et al., 2017), and ssPCA (Liu, 2020). Most of these are nonlinear methods. Newer linear dimensionality reduction methods in processing scRNA-seq datasets are scarce. A linear transformation of the original data matrix leads to separation and stretching of the raw data. The advantage of it is that the linear relationship between variables in the original data can be preserved. Therefore, we propose a linear method, nPCA, to upgrade PCA.

nPCA outperforms PCA by using the deep learning method. PCA calculates the projection matrix through SVD at one time, while nPCA continuously corrects the projection matrix through SGD in the backpropagation algorithm. Furthermore, nPCA did not perform well in public datasets (like blood transfusion service center and seeds, Table 2), but the performance gap is not large compared with PCA. The reason could be that the projection direction with the largest variance in these datasets is the direction that contains the most information. In addition, nPCA, like PCA, is also sensitive to outliers and missing values.

In scRNA-seq datasets, the losses of nPCA are all better than those of PCA. Due to big data, nPCA has sufficient training samples to achieve the best results. However, because of the existence of the neural network, nPCA is time-consuming to a certain extent. At the cost of time-consuming operations, nPCA achieves a good dimensionality reduction effect. Figure 2 and Figure 3 show that nPCA has the ability to separate the categories that are mixed together in other methods.

## 5 Conclusion

In this study, we introduced nPCA, which is a novel linear dimensionality reduction technique using a multilayer perceptron. nPCA outperformed traditional PCA and similar methods in our extensive tests across various datasets, particularly in single-cell RNA sequencing data, where it demonstrated superior variance capture and clustering capabilities. Our work demonstrated that it is time to consider a modernization of PCA with the advances in the field of science and technology, and it offers a valuable tool for biological data analysis that combines deep learning benefits with linear methods’ simplicity and interpretability.

## Data Availability

The original contributions presented in the study are included in the article/Supplementary Material; further inquiries can be directed to the corresponding author.
