# Supplementary material for: nPCA: a linear dimensionality reduction method using a multilayer perceptron
Source: Front Genet. 2024 Jan 8;14:1290447. doi: 10.3389/fgene.2023.1290447 (PMC10800564; doi:10.3389/fgene.2023.1290447)
Supplement: Supplementary file 1 [file Table1.DOCX]

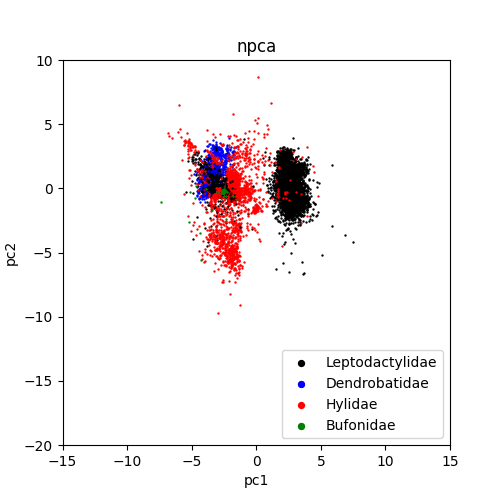


**Gif S1.** The training process in nPCA of Anuran calls (MFCCs) which include 90 pictures. The most notable change is in the first ten pictures


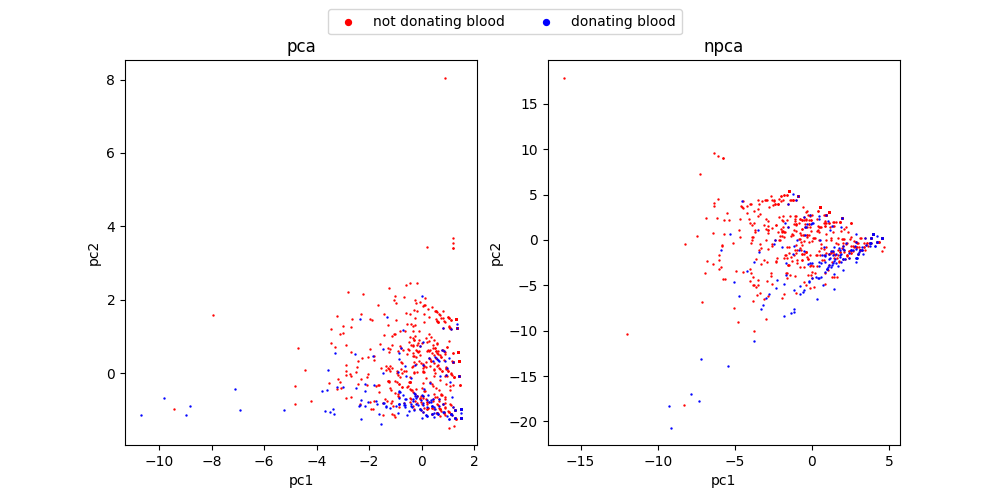


**Figure S1.** Two-dimensional PCA and nPCA projection of Blood Transfusion Service Center dataset colored by real label (whether he/she donated blood in March 2007)


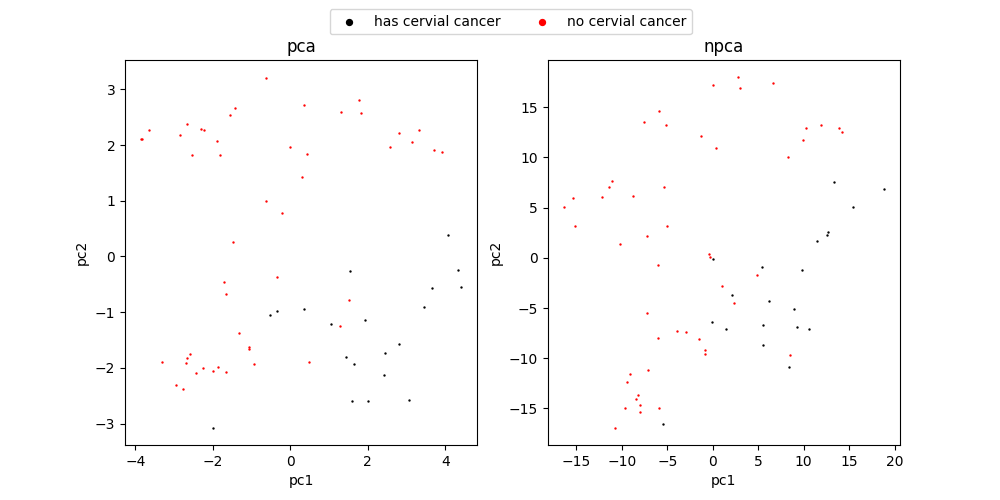


**Figure S2.** Two-dimensional PCA and nPCA projection of Cervical Cancer Behavior Risk dataset colored by real label (the respondent with or without ca cervix).


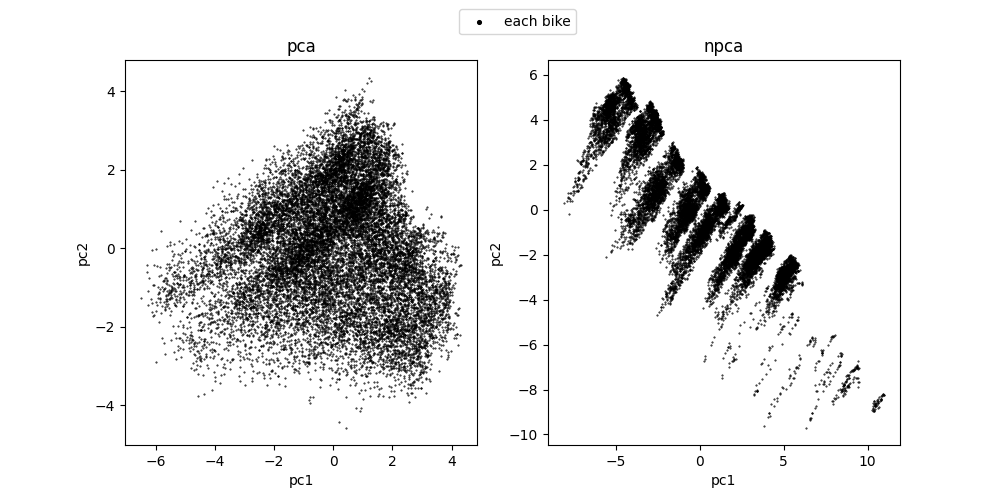


**Figure S3.** Two-dimensional PCA and nPCA projection of Bike dataset (The associated task of this dataset is regression, so no classes were given)


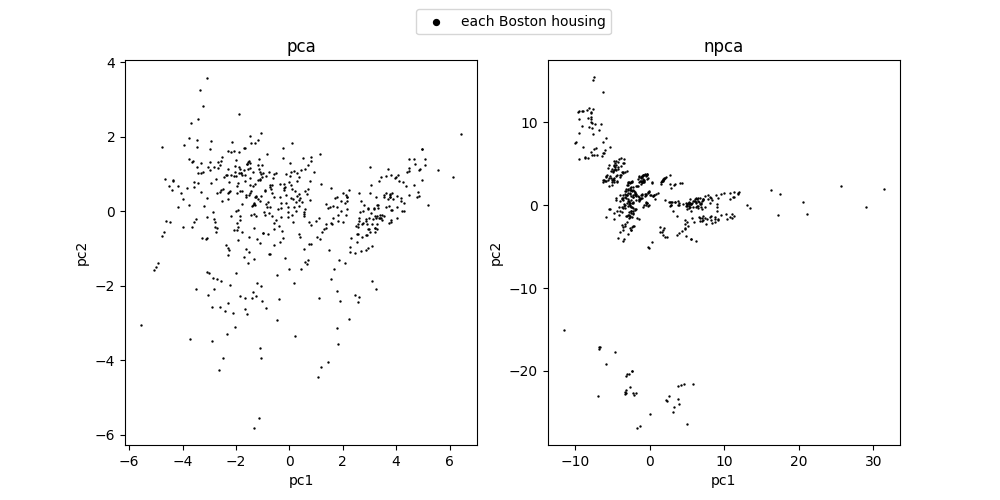


**Figure S4.** Two-dimensional PCA and nPCA projection of Housing dataset (The associated task of this dataset is regression, so no classes were given)


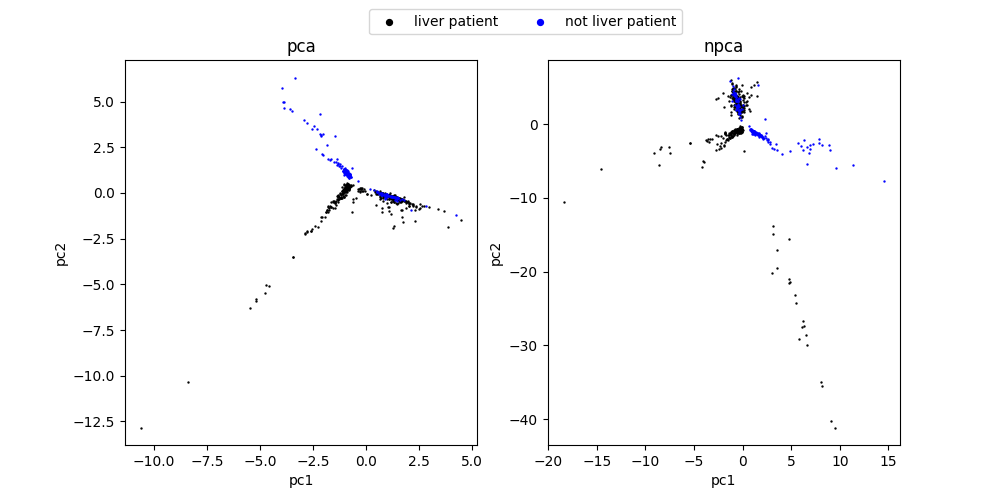


**Figure S5.** Two-dimensional PCA and nPCA projection of Indian Liver Patient dataset colored by real label (the patient with or without liver disease)


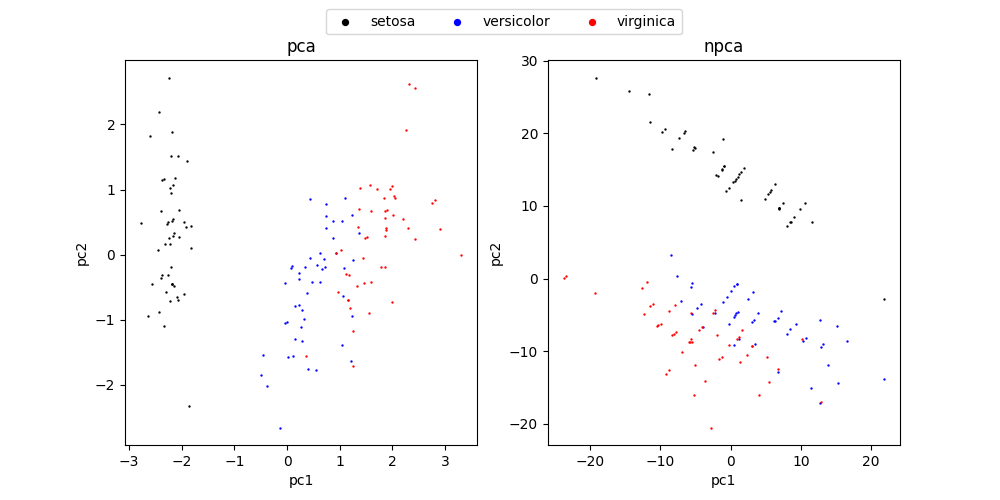


**Figure S6.** Two-dimensional PCA and nPCA projection of iris dataset colored by real label (type of iris plant).


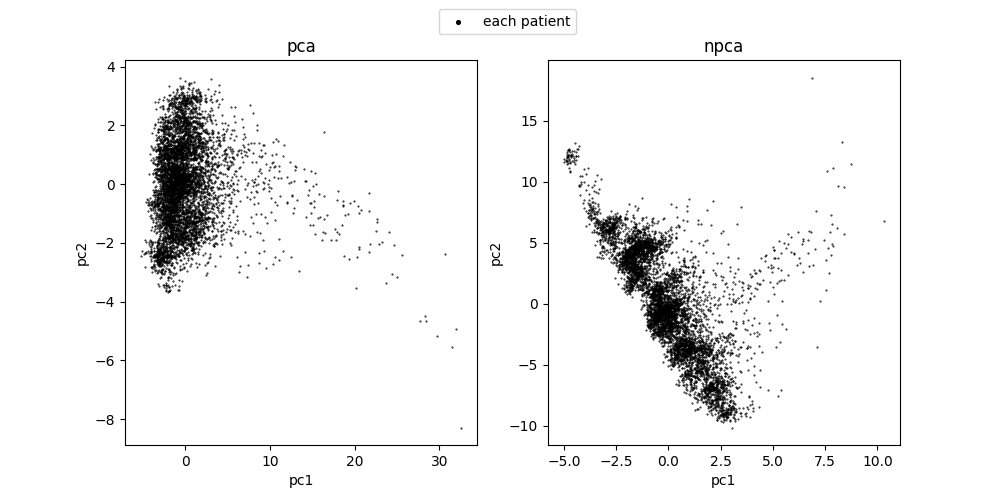


**Figure S7.** Two-dimensional PCA and nPCA projection of Parkinsons Telemonitoring dataset (The associated task of this dataset is regression, so no classes were given)


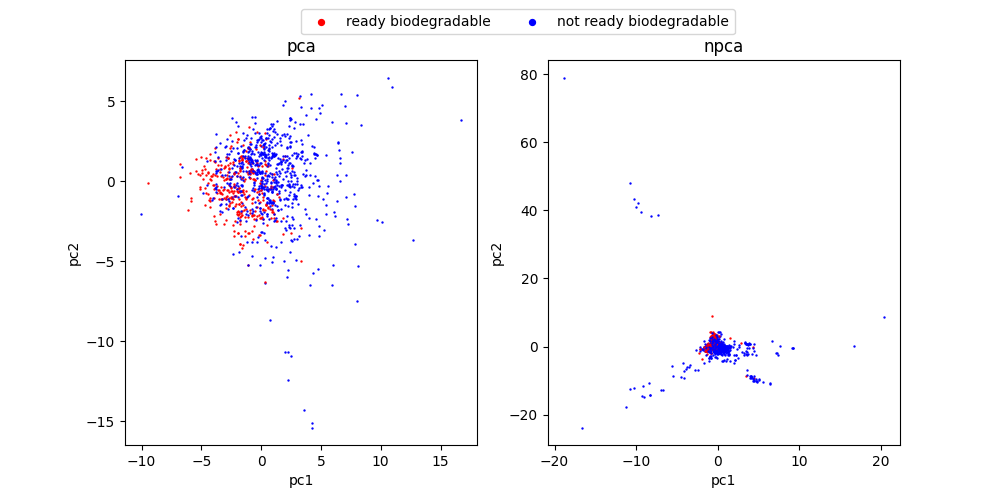


**Figure S8.** Two-dimensional PCA and nPCA projection of QSAR Biodegradation dataset colored by real label (ready biodegradable and not ready biodegradable)


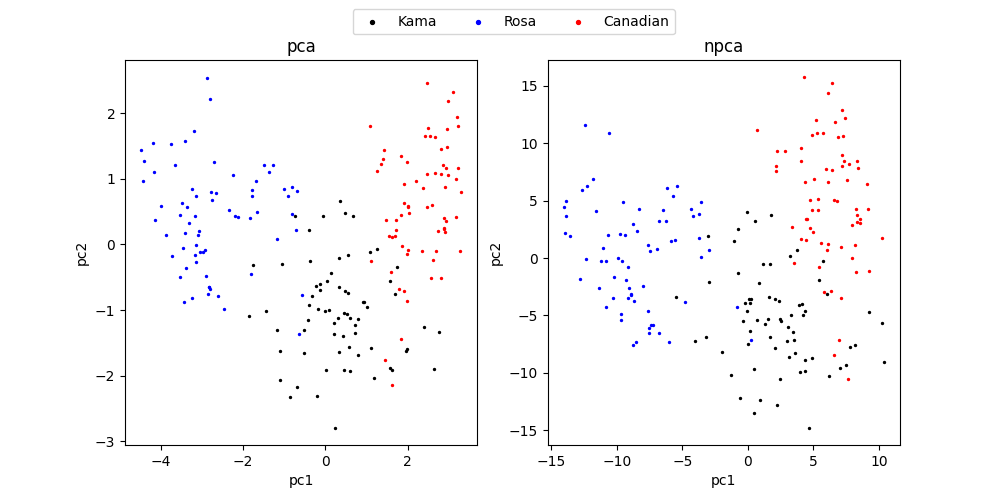


**Figure S9.** Two-dimensional PCA and nPCA projection of Seeds dataset colored by real label (three different varieties of wheat: Kama, Rosa and Canadian).


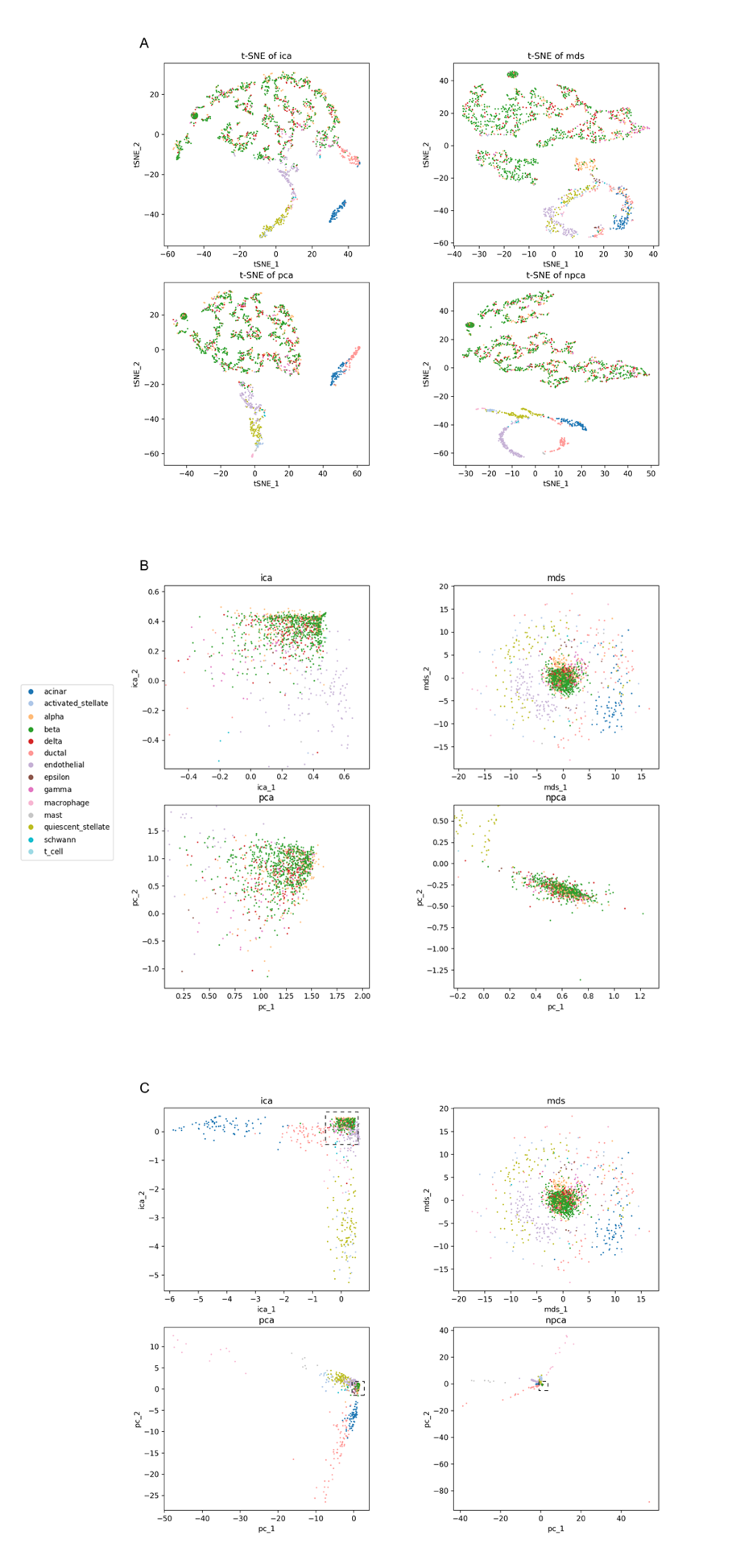


**Figure S10.** visualization for Baron human1 of 200 highly variable genes (A): comparison of t-SNE of four linear methods, (B): partially enlarged view of (C), (C): comparison of four linear methods. Each point represents a cell and is colored by the real label.


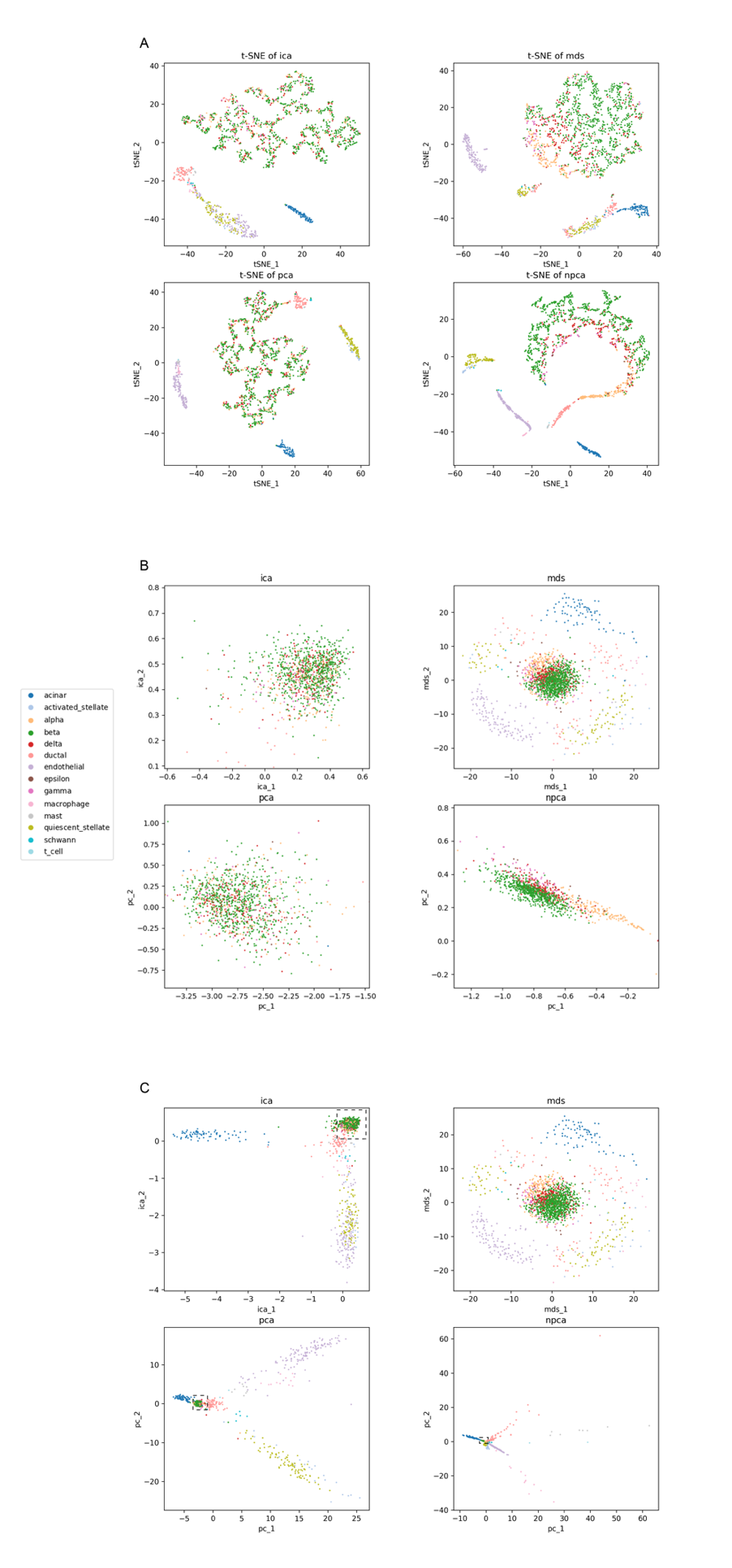


**Figure S11.** visualization for Baron human1 of 500 highly variable genes (A): comparison of t-SNE of four linear methods, (B): partially enlarged view of (C), (C): comparison of four linear methods. Each point represents a cell and is colored by the real label.


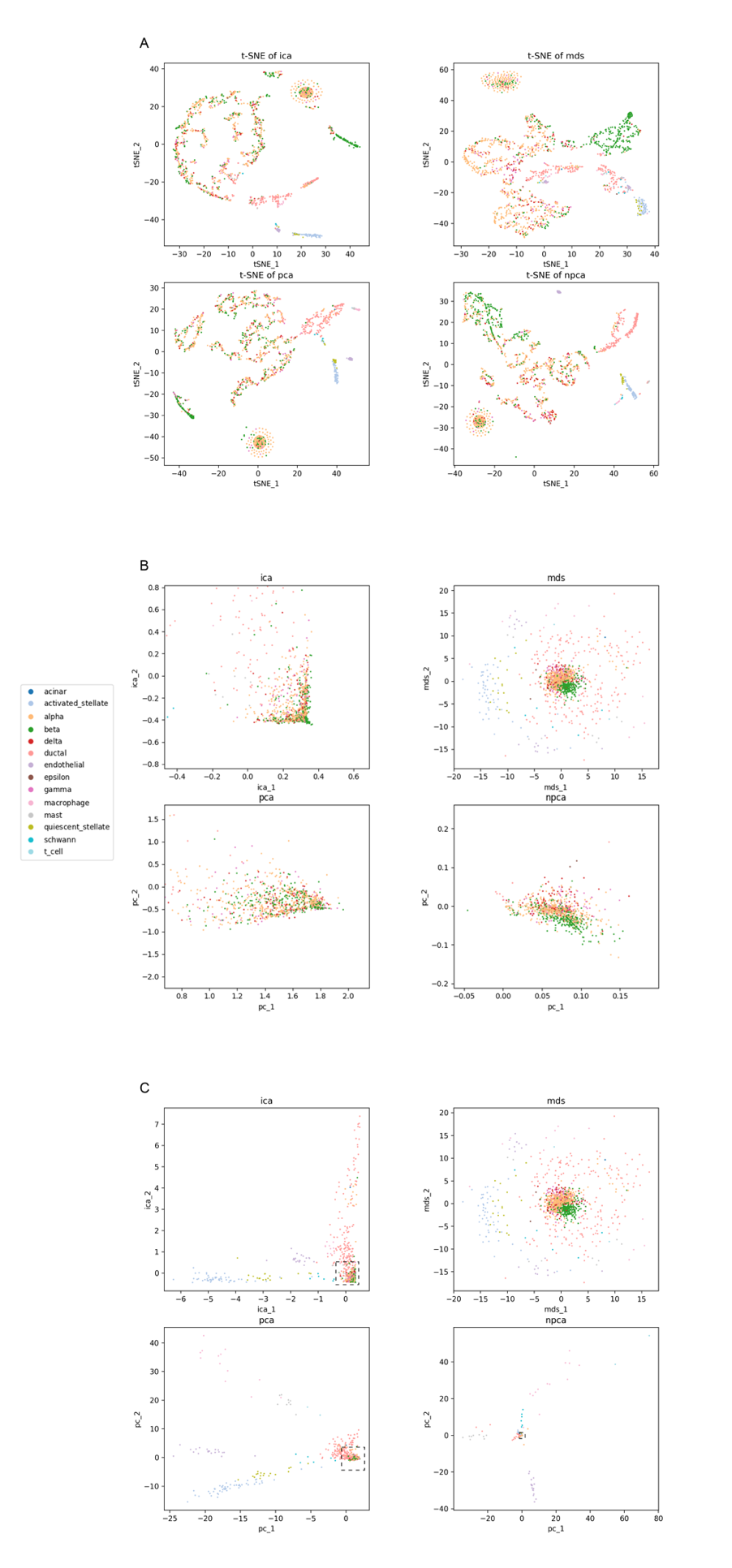


**Figure S12.** visualization for Baron human2 of 200 highly variable genes (A): comparison of t-SNE of four linear methods, (B): partially enlarged view of (C), (C): comparison of four linear methods. Each point represents a cell and is colored by the real label.


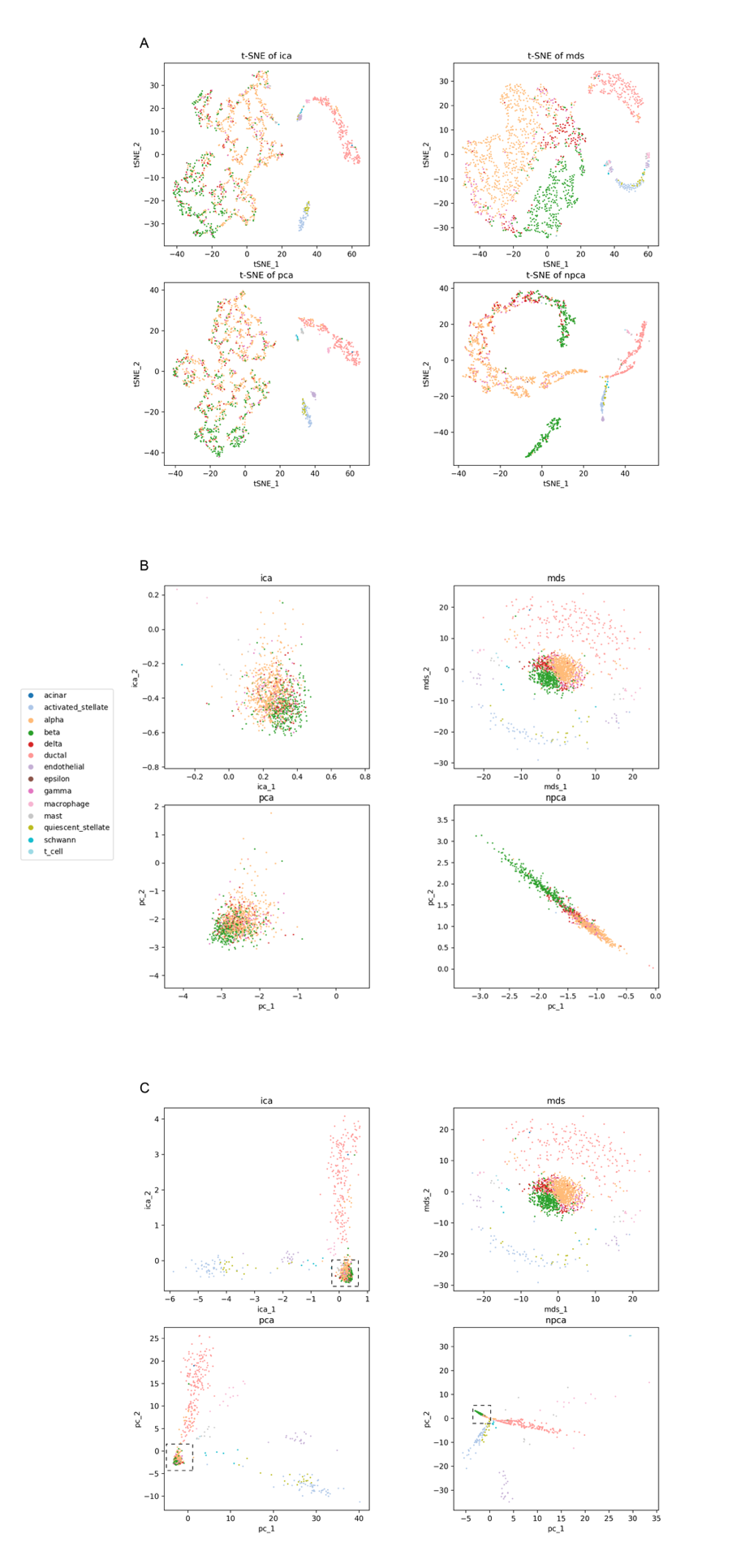


**Figure S13.** visualization for Baron human2 of 500 highly variable genes (A): comparison of t-SNE of four linear methods, (B): partially enlarged view of (C), (C): comparison of four linear methods. Each point represents a cell and is colored by the real label.


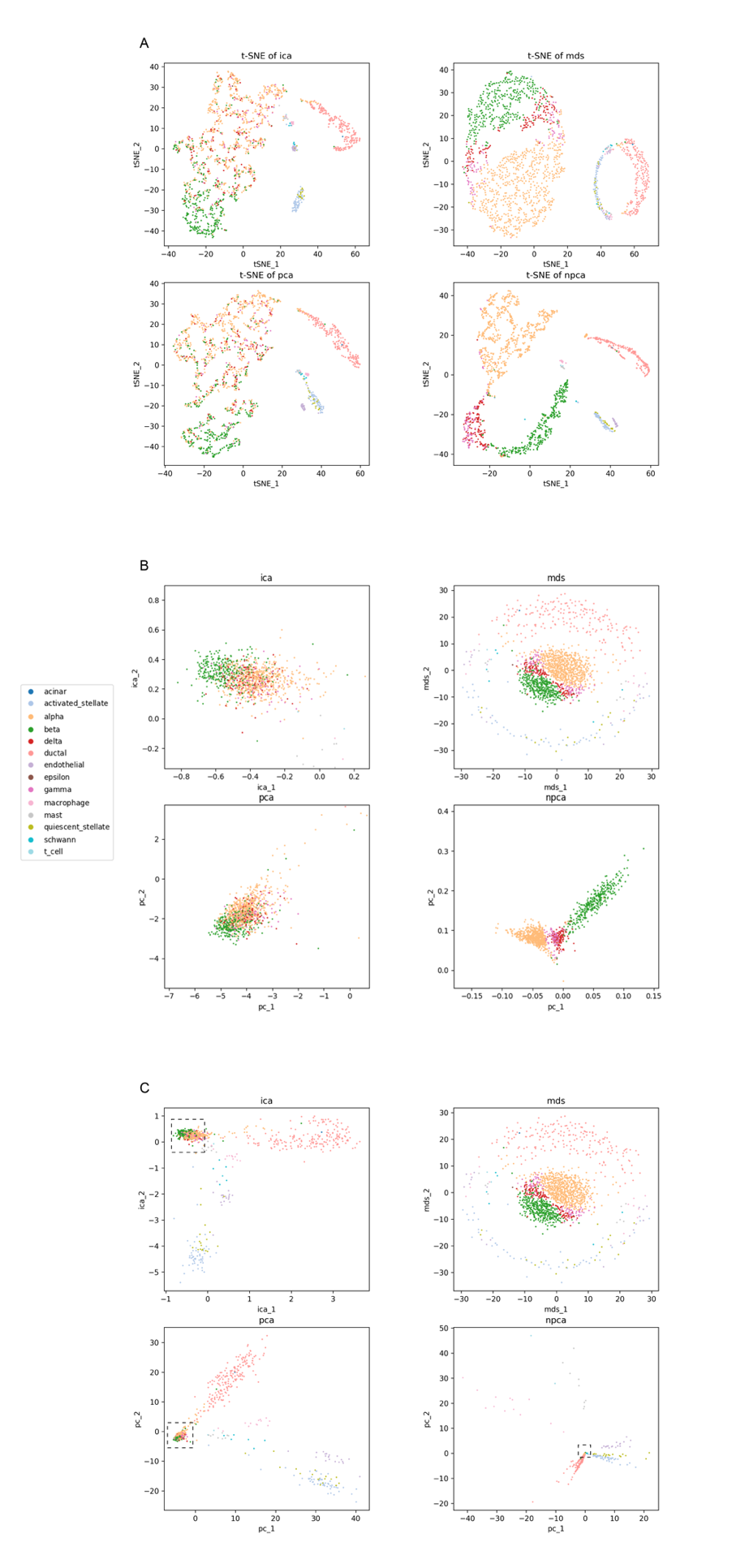


**Figure S14.** visualization for Baron human2 of 1000 highly variable genes (A): comparison of t-SNE of four linear methods, (B): partially enlarged view of (C), (C): comparison of four linear methods. Each point represents a cell and is colored by the real label.


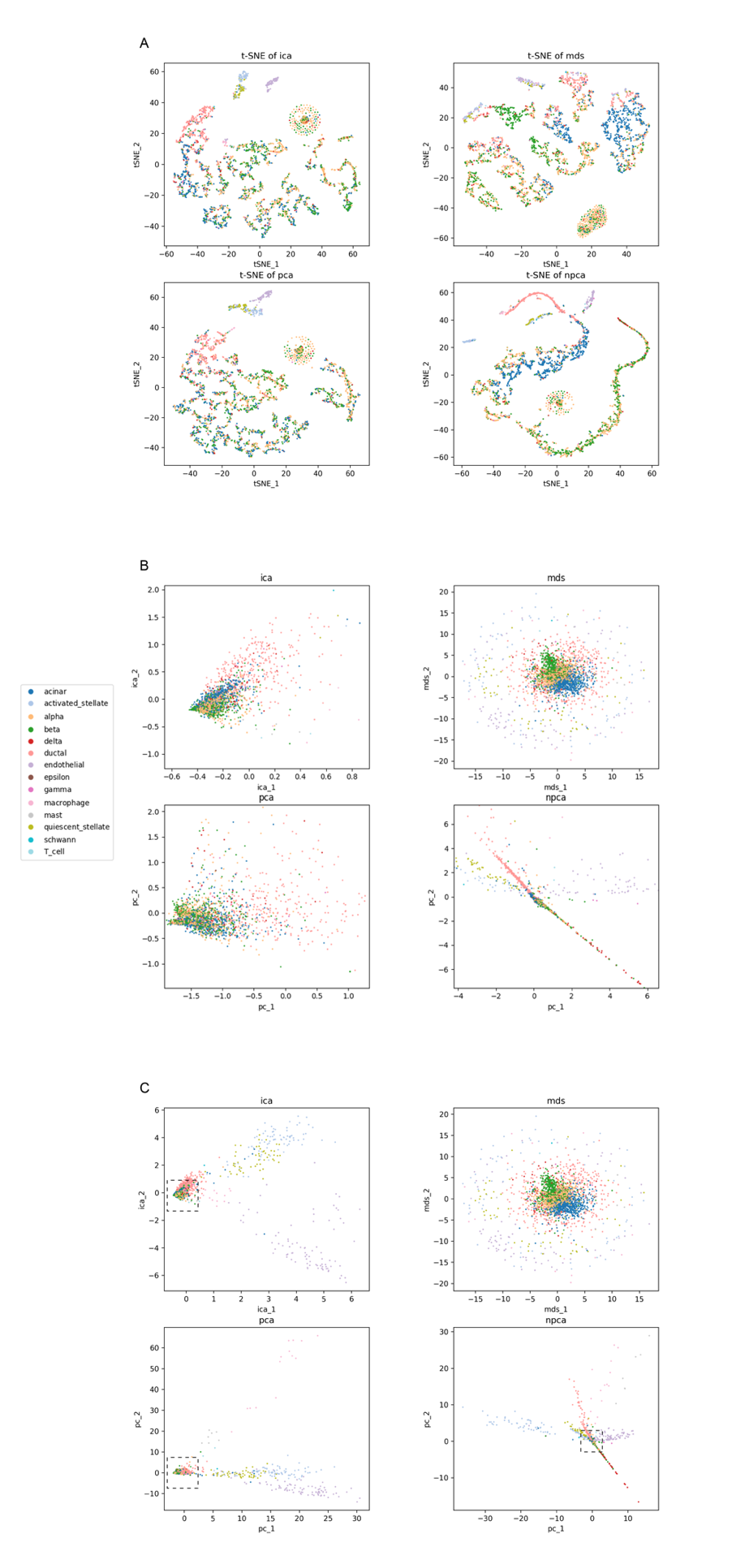


**Figure S15.** visualization for Baron human3 of 200 highly variable genes (A): comparison of t-SNE of four linear methods, (B): partially enlarged view of (C), (C): comparison of four linear methods. Each point represents a cell and is colored by the real label.


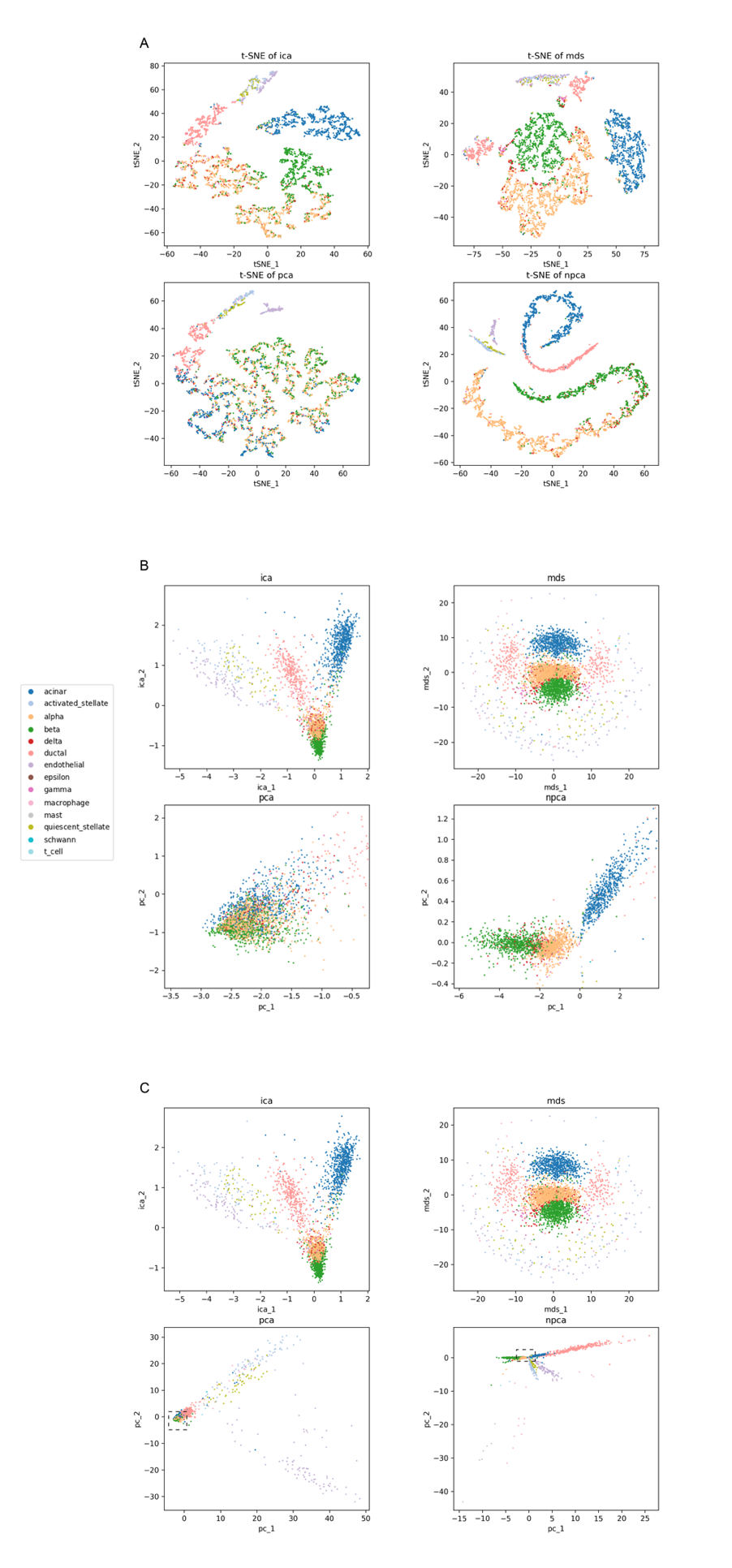


**Figure S16.** visualization for Baron human3 of 500 highly variable genes (A): comparison of t-SNE of four linear methods, (B): partially enlarged view of (C), (C): comparison of four linear methods. Each point represents a cell and is colored by the real label.


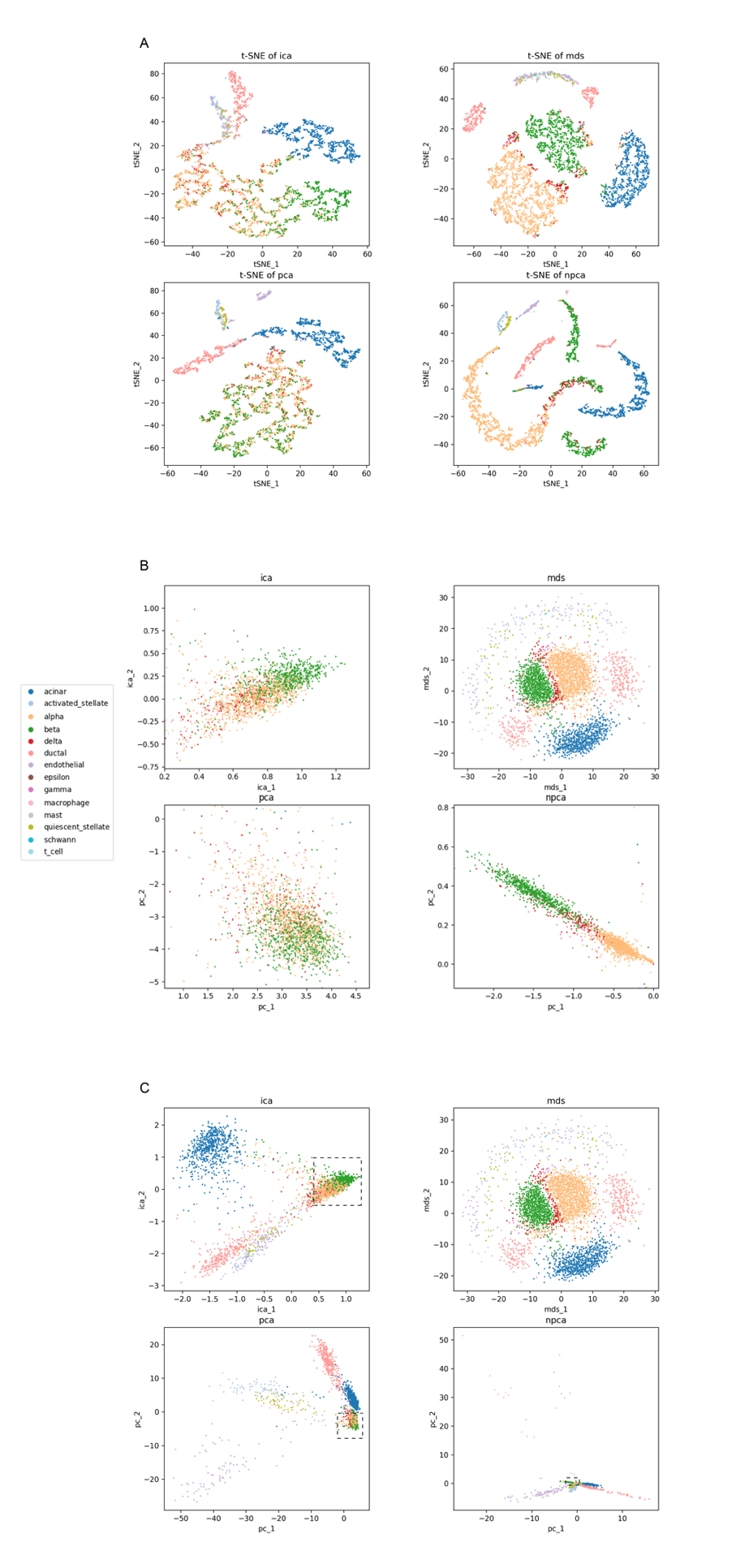


**Figure S17.** visualization for Baron human3 of 1000 highly variable genes (A): comparison of t-SNE of four linear methods, (B): partially enlarged view of (C), (C): comparison of four linear methods. Each point represents a cell and is colored by the real label.


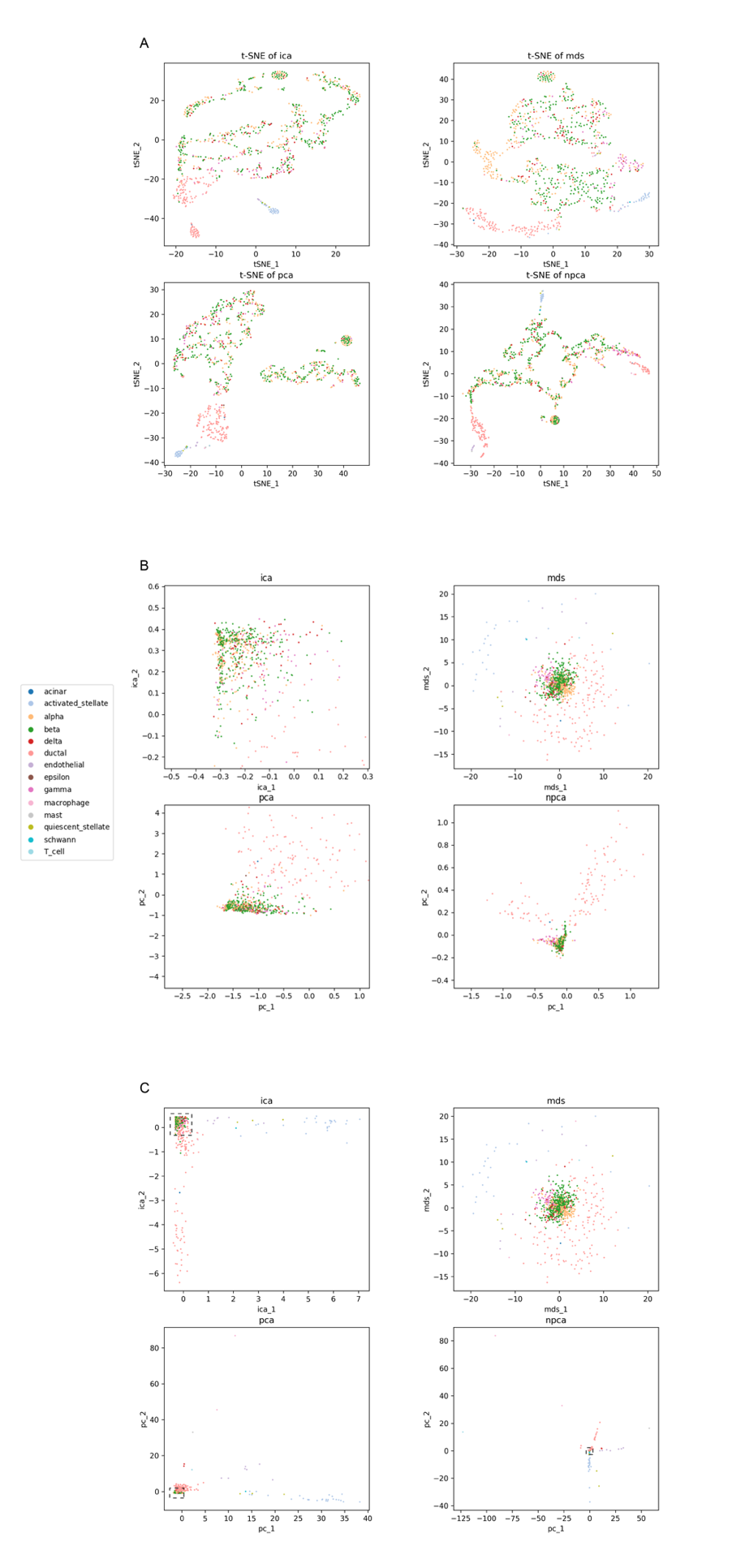


**Figure S18.** visualization for Baron human4 of 200 highly variable genes (A): comparison of t-SNE of four linear methods, (B): partially enlarged view of (C), (C): comparison of four linear methods. Each point represents a cell and is colored by the real label.


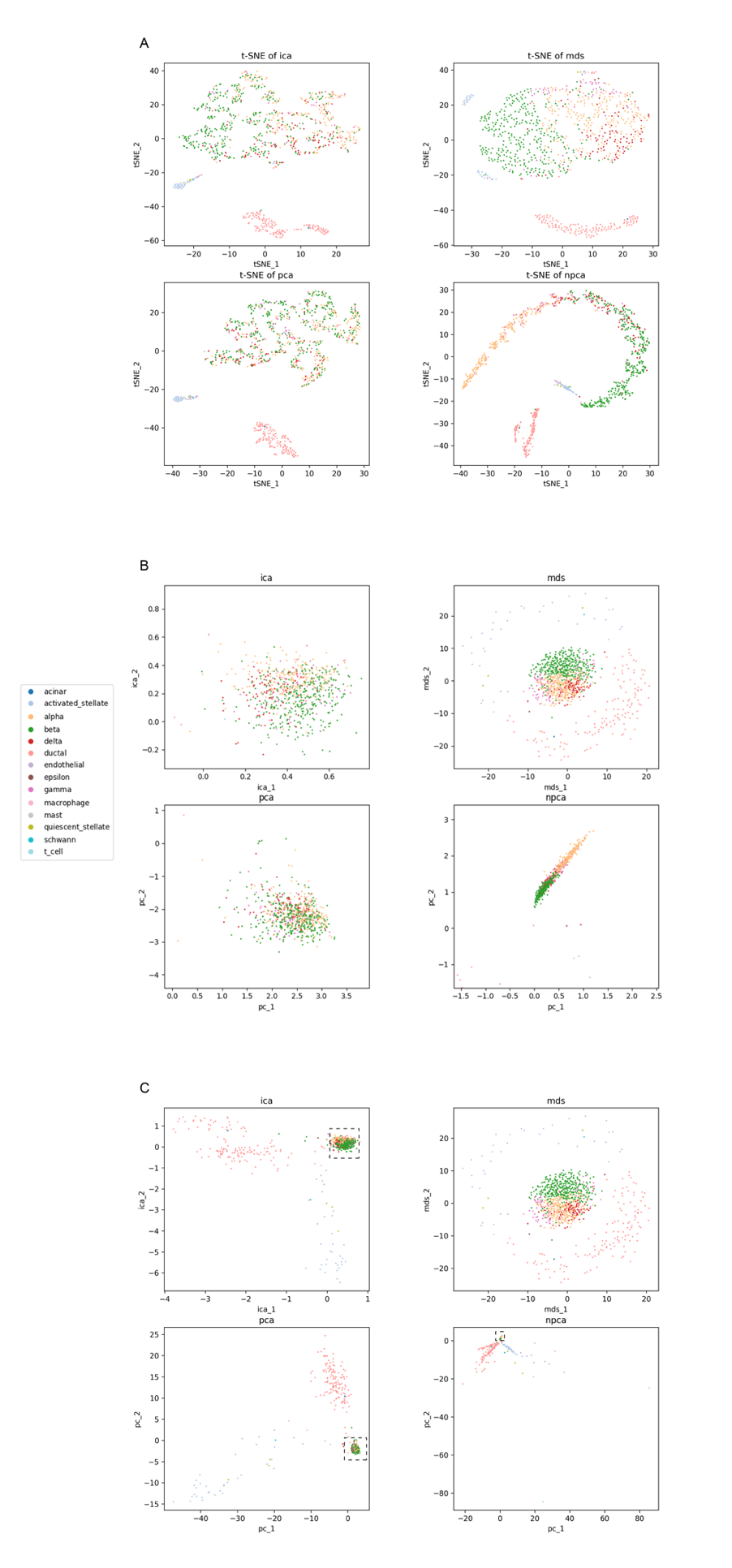


**Figure S19.** visualization for Baron human4 of 500 highly variable genes (A): comparison of t-SNE of four linear methods, (B): partially enlarged view of (C), (C): comparison of four linear methods. Each point represents a cell and is colored by the real label.


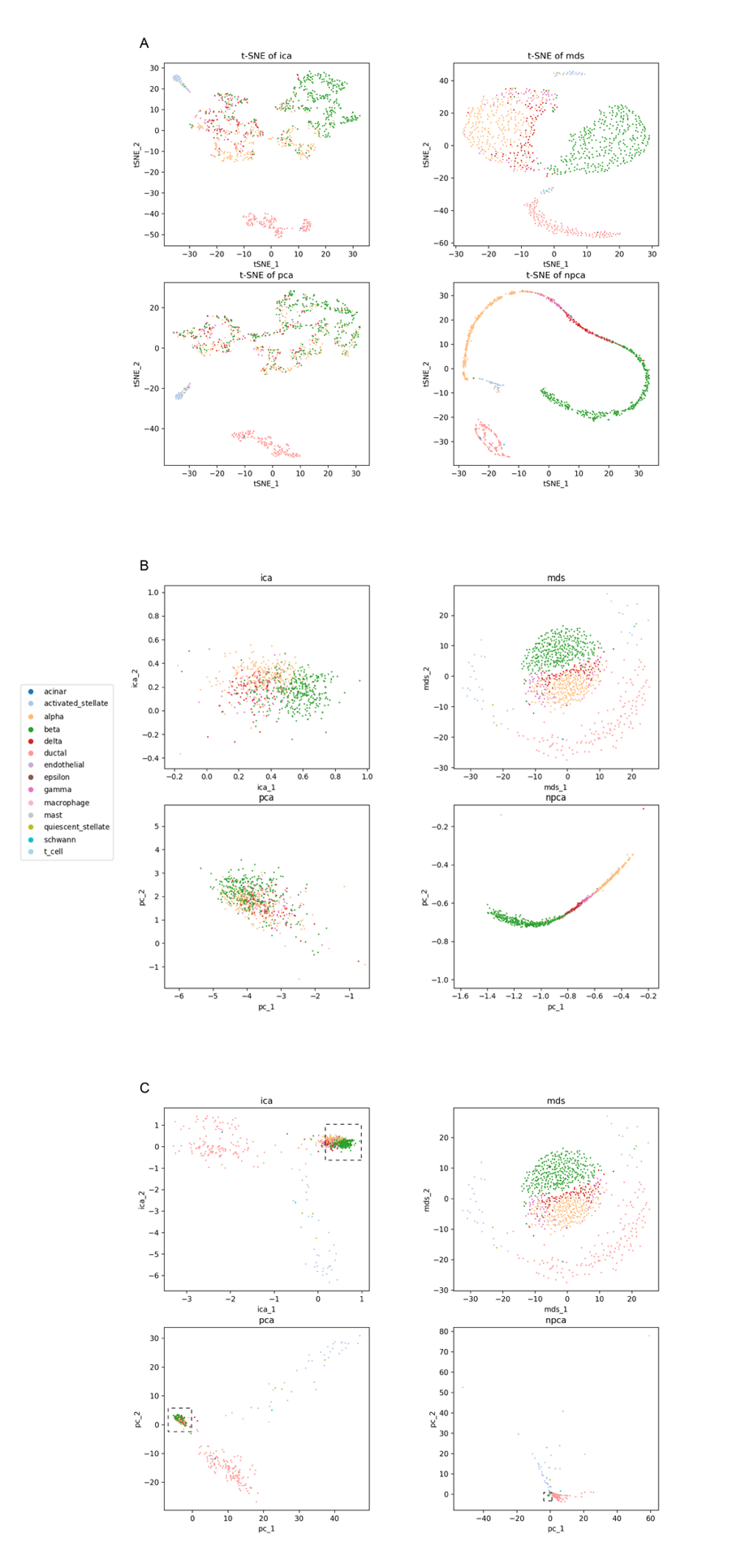


**Figure S20.** visualization for Baron human4 of 1000 highly variable genes (A): comparison of t-SNE of four linear methods, (B): partially enlarged view of (C), (C): comparison of four linear methods. Each point represents a cell and is colored by the real label.


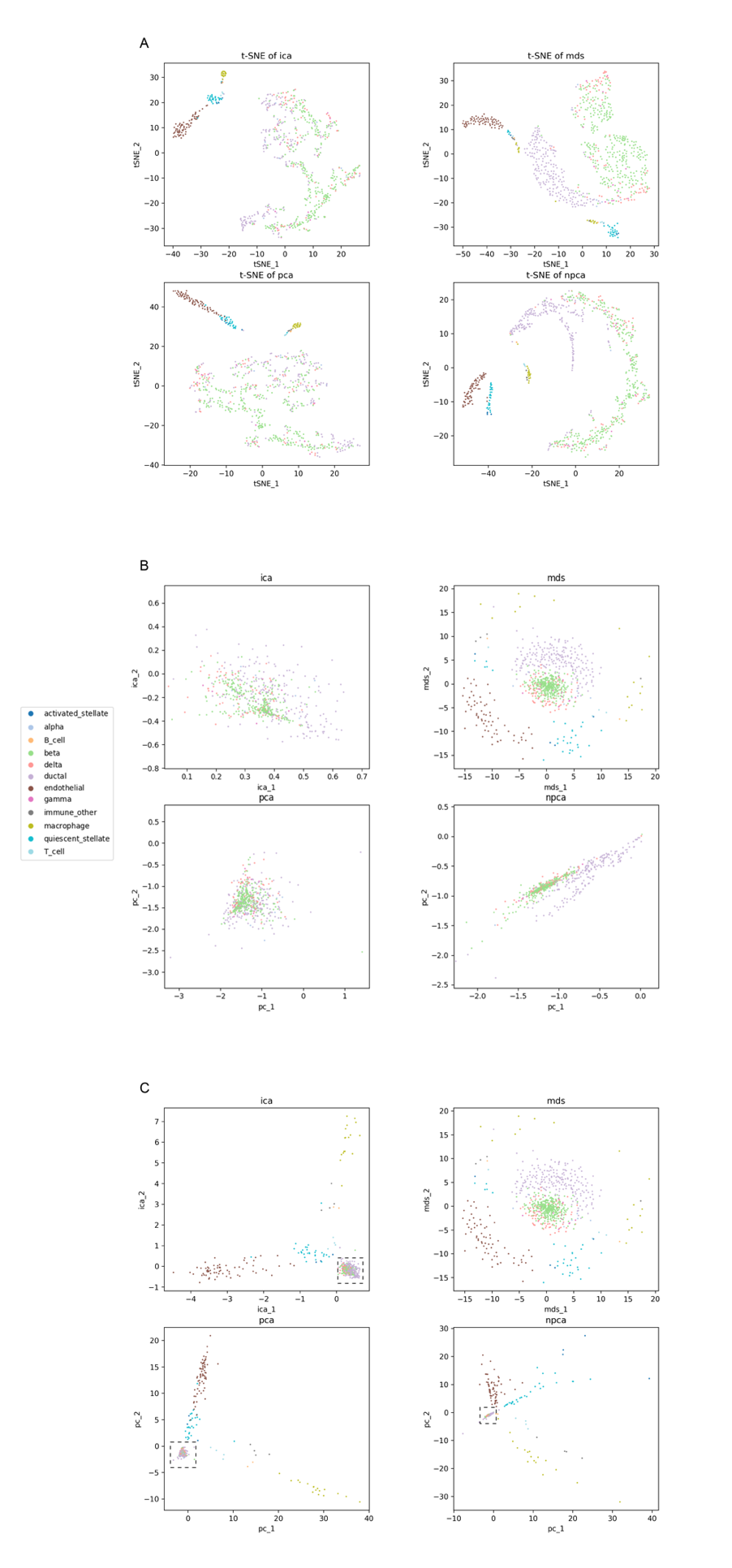


**Figure S21.** visualization for Baron mouse1 of 200 highly variable genes (A): comparison of t-SNE of four linear methods, (B): partially enlarged view of (C), (C): comparison of four linear methods. Each point represents a cell and is colored by the real label.


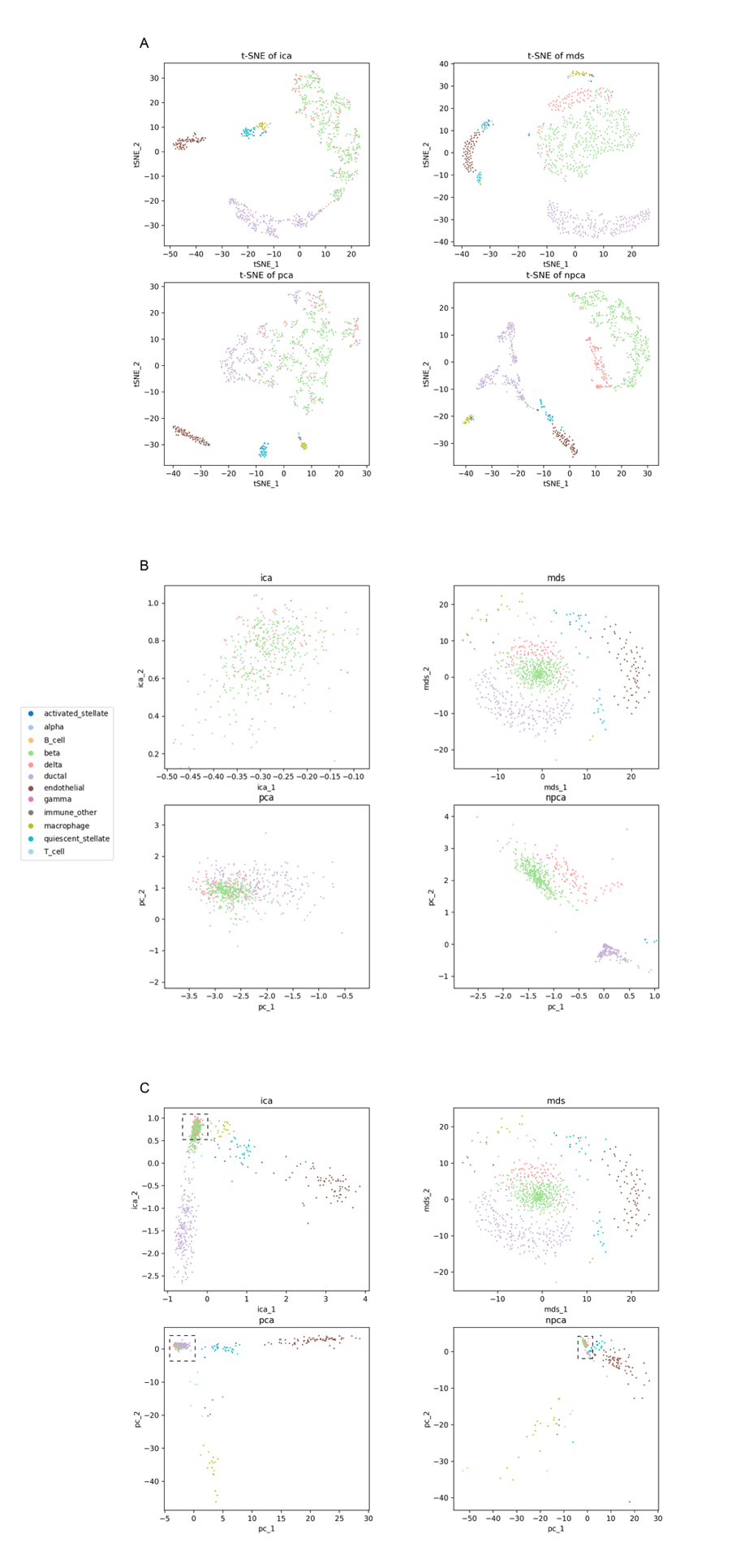


**Figure S22.** visualization for Baron mouse1 of 500 highly variable genes (A): comparison of t-SNE of four linear methods, (B): partially enlarged view of (C), (C): comparison of four linear methods. Each point represents a cell and is colored by the real label.


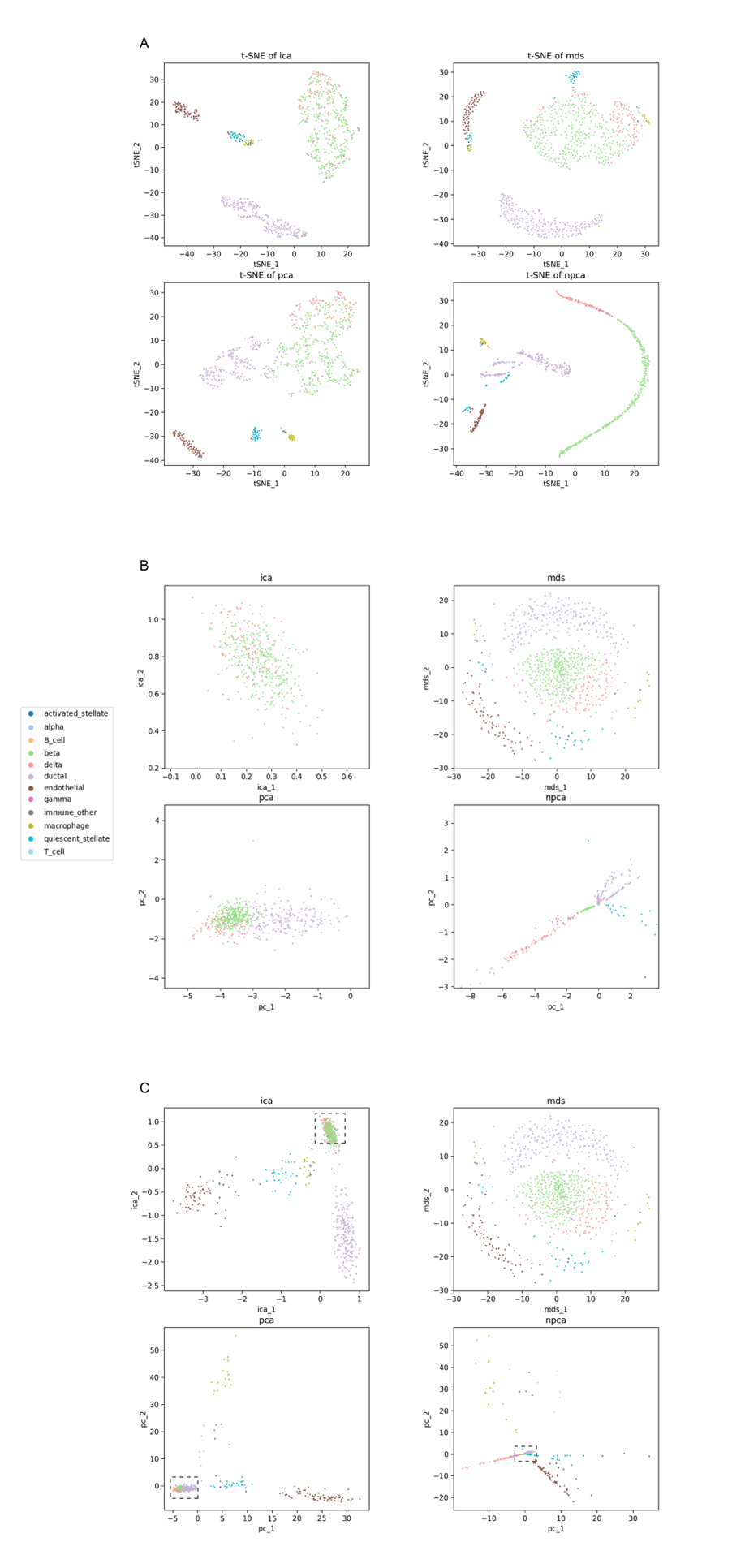


**Figure S23.** visualization for Baron mouse1 of 1000 highly variable genes (A): comparison of t-SNE of four linear methods, (B): partially enlarged view of (C), (C): comparison of four linear methods. Each point represents a cell and is colored by the real label.


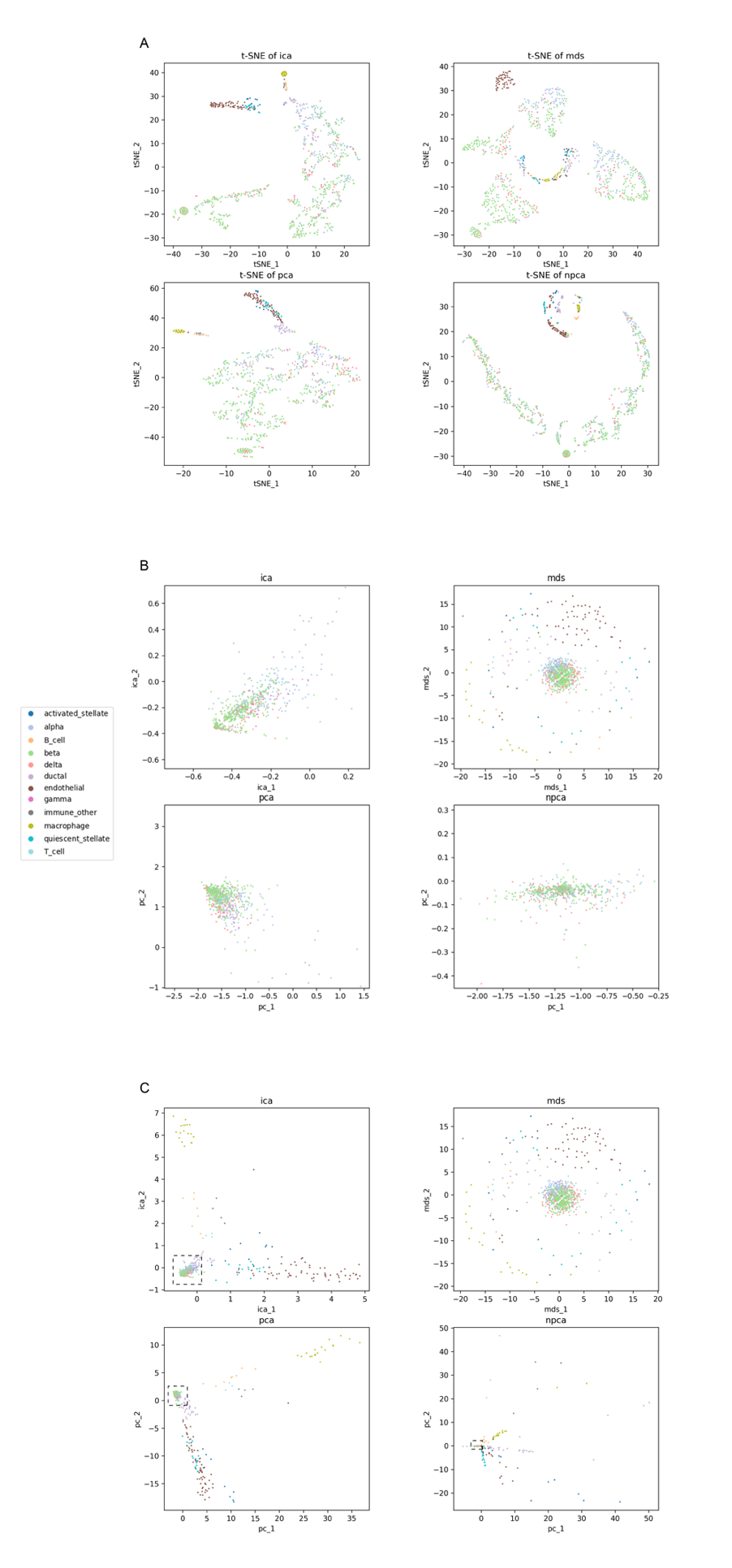


**Figure S24.** visualization for Baron mouse2 of 200 highly variable genes (A): comparison of t-SNE of four linear methods, (B): partially enlarged view of (C), (C): comparison of four linear methods. Each point represents a cell and is colored by the real label.


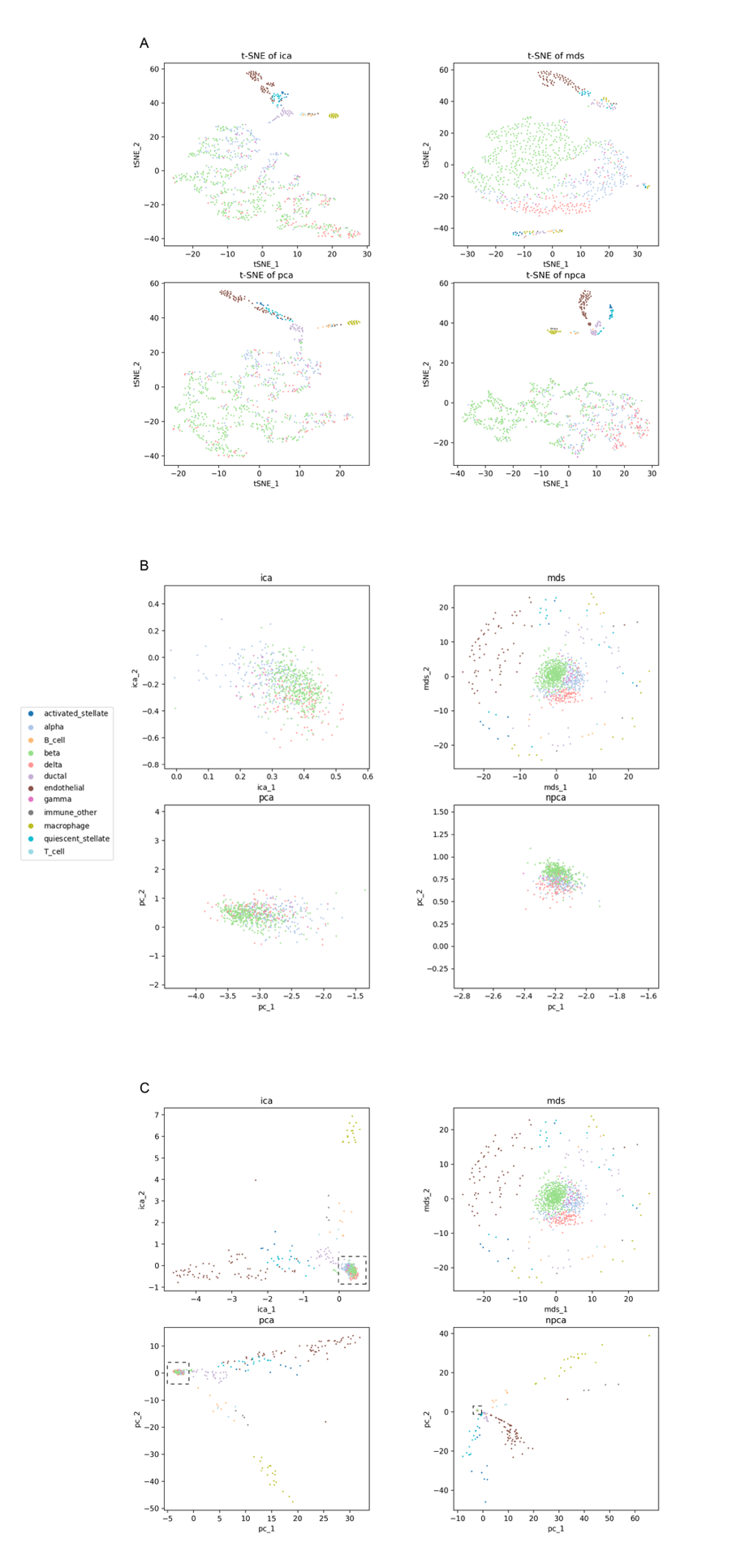


**Figure S25.** visualization for Baron mouse2 of 500 highly variable genes (A): comparison of t-SNE of four linear methods, (B): partially enlarged view of (C), (C): comparison of four linear methods. Each point represents a cell and is colored by the real label.


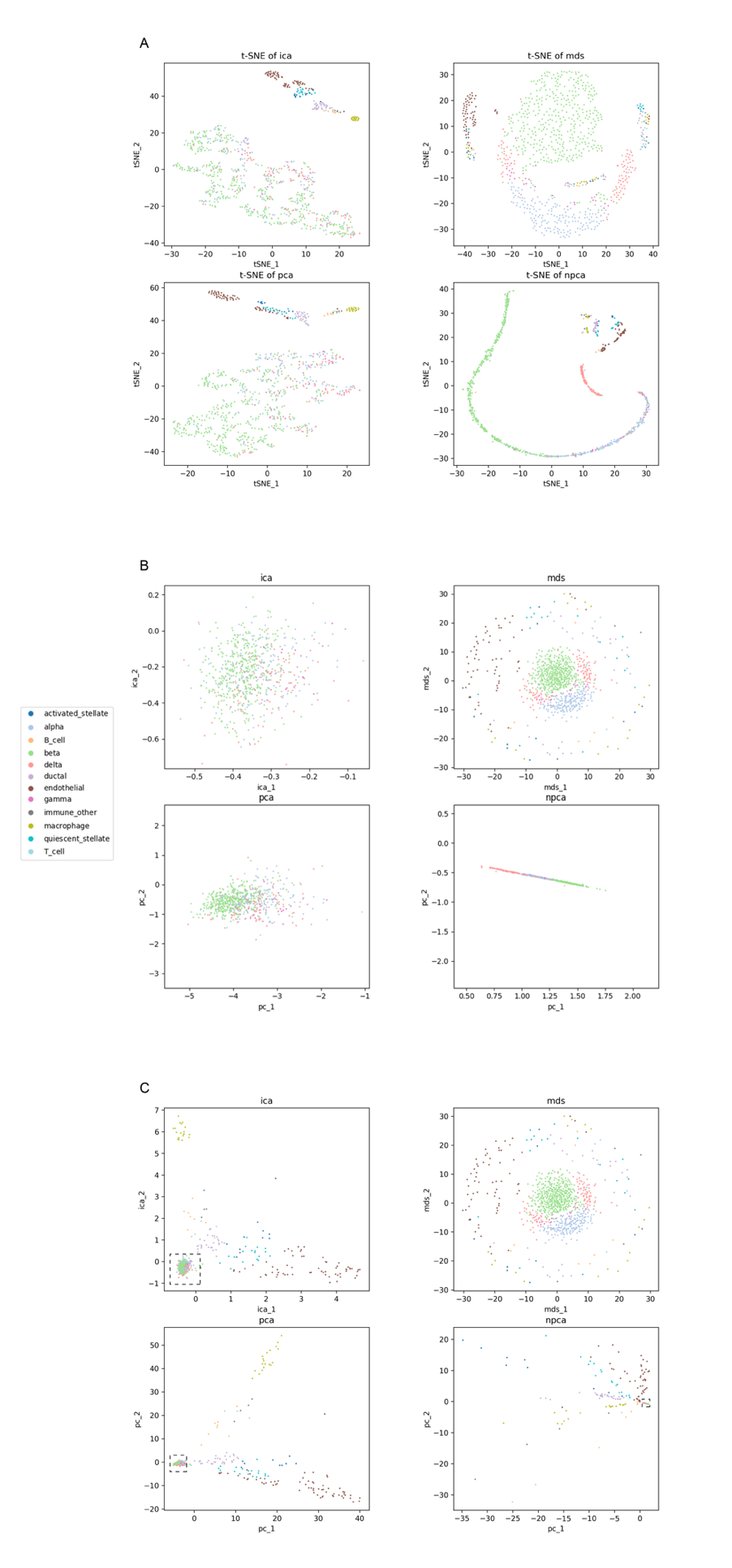


**Figure S26.** visualization for Baron mouse2 of 1000 highly variable genes (A): comparison of t-SNE of four linear methods, (B): partially enlarged view of (C), (C): comparison of four linear methods. Each point represents a cell and is colored by the real label.


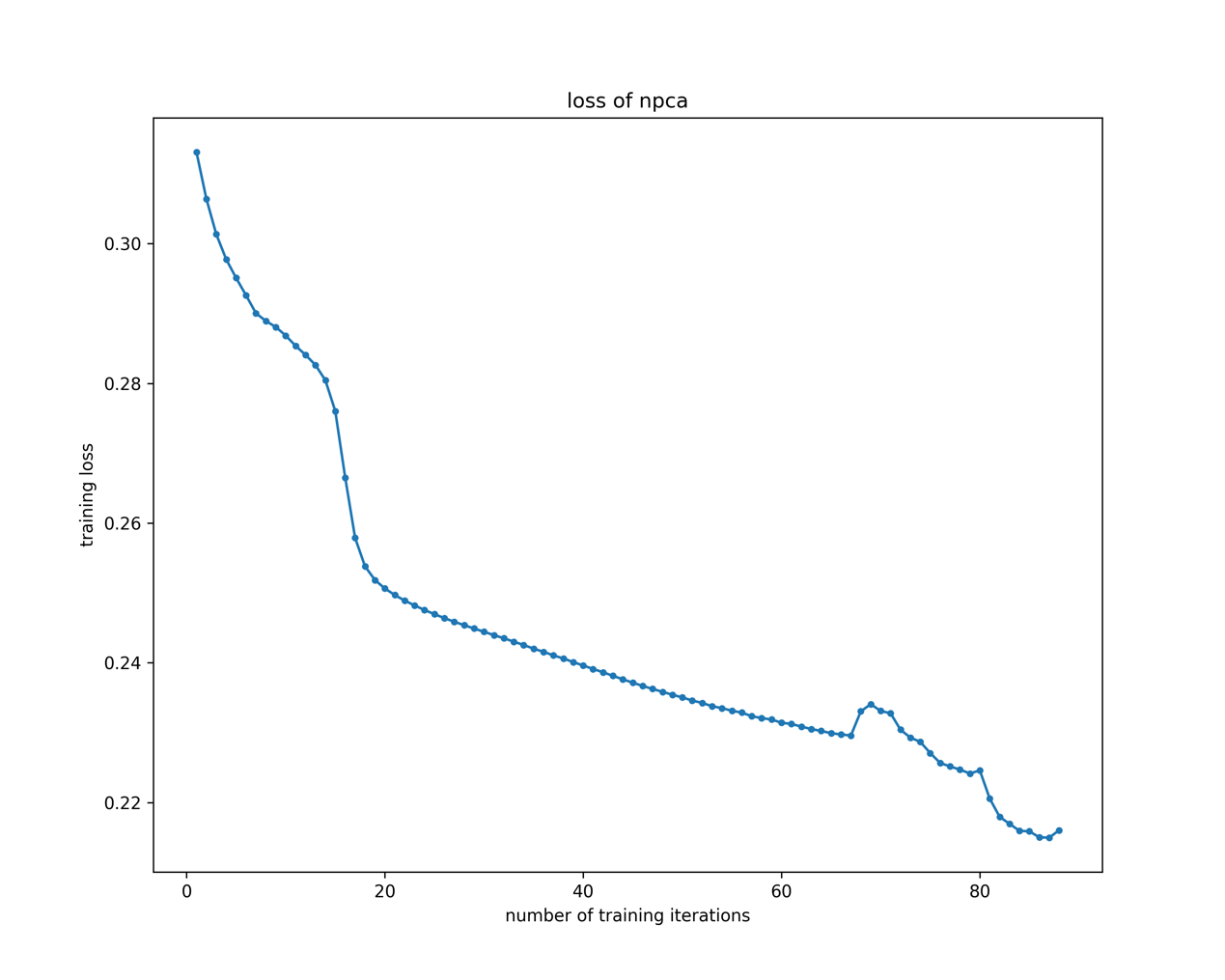


**Figure S27.** the loss of nPCA of bike dataset


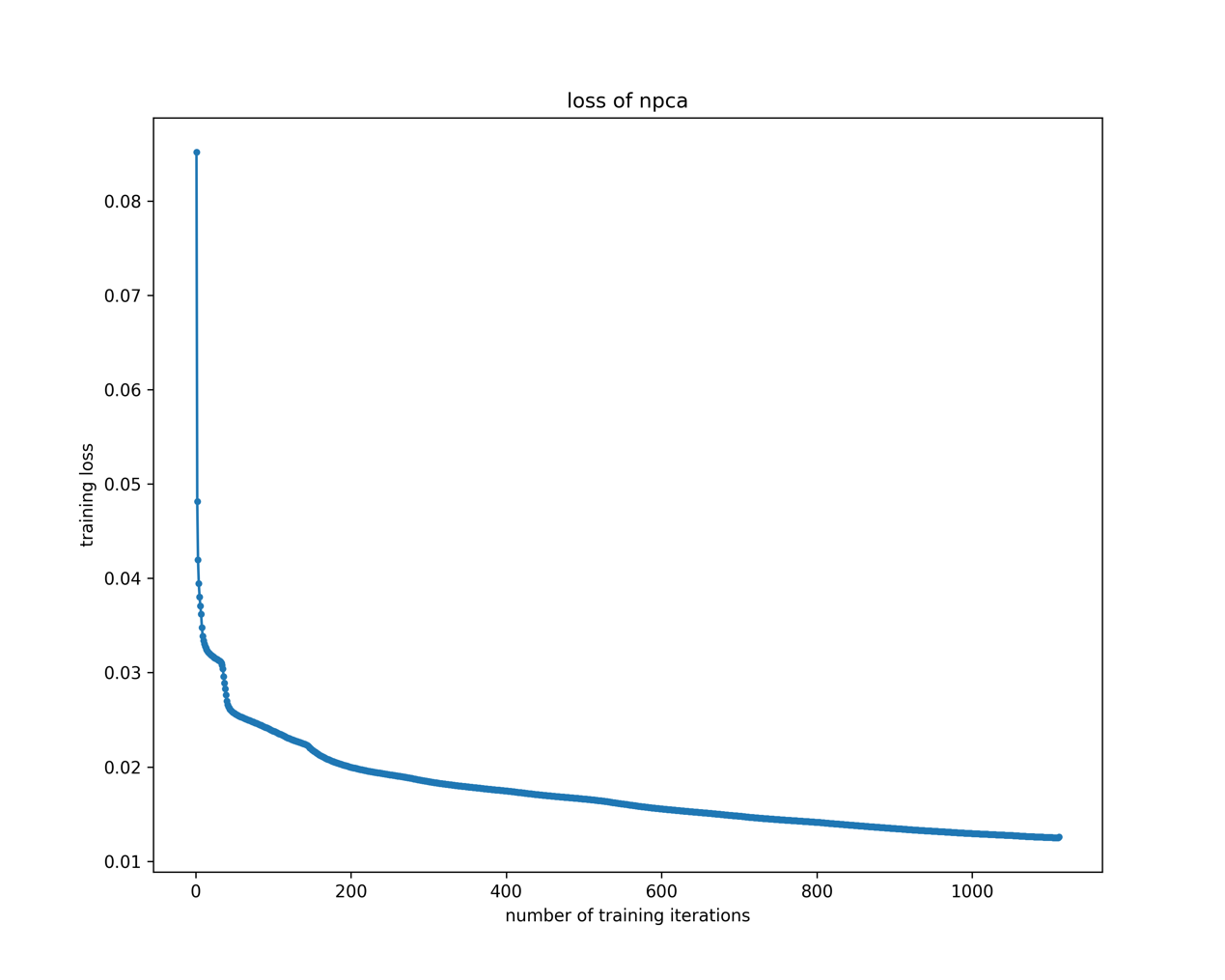


**Figure S28.** the loss of nPCA of blood transfusion service center dataset


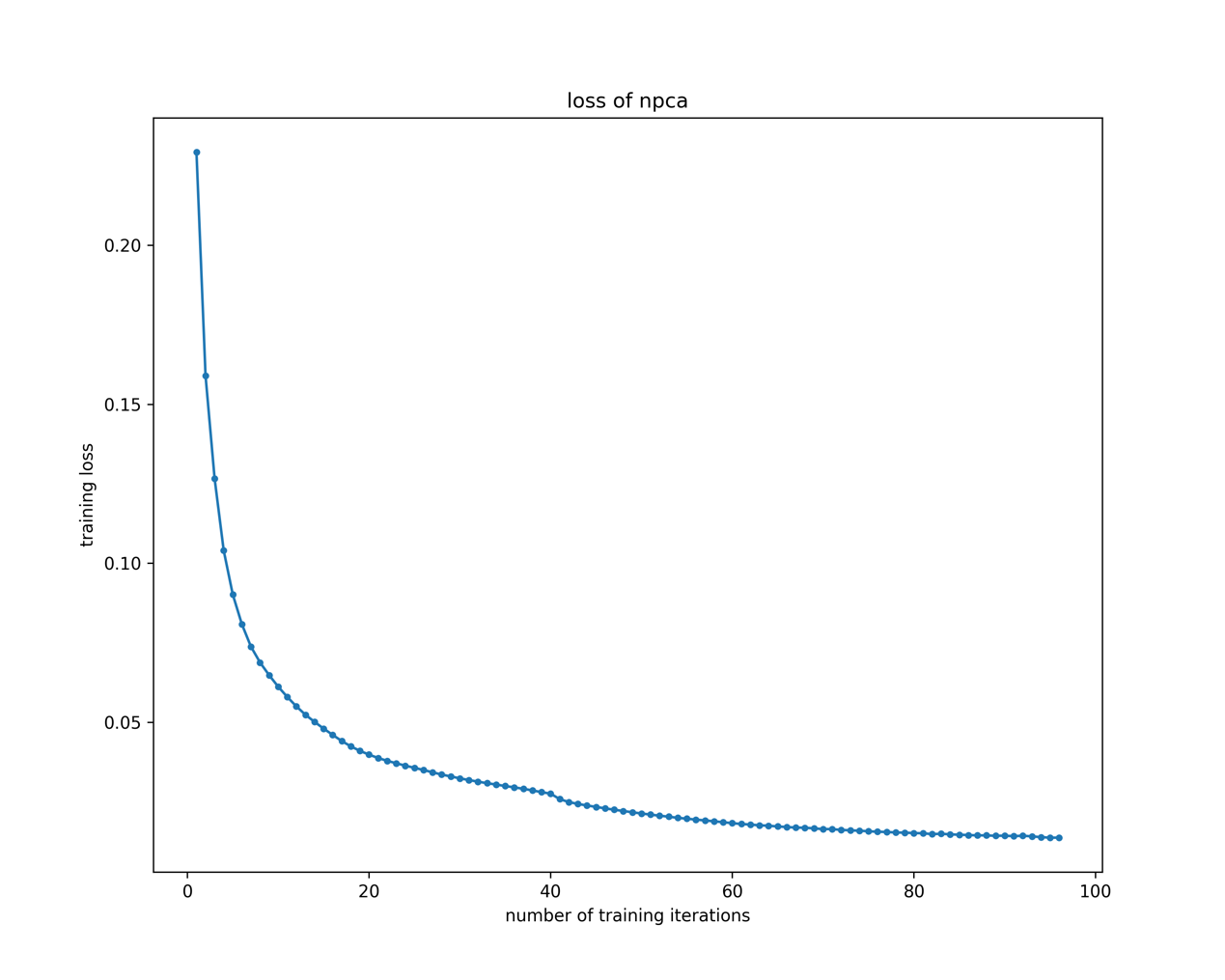


**Figure S29.** the loss of nPCA of cervical cancer behavior risk dataset


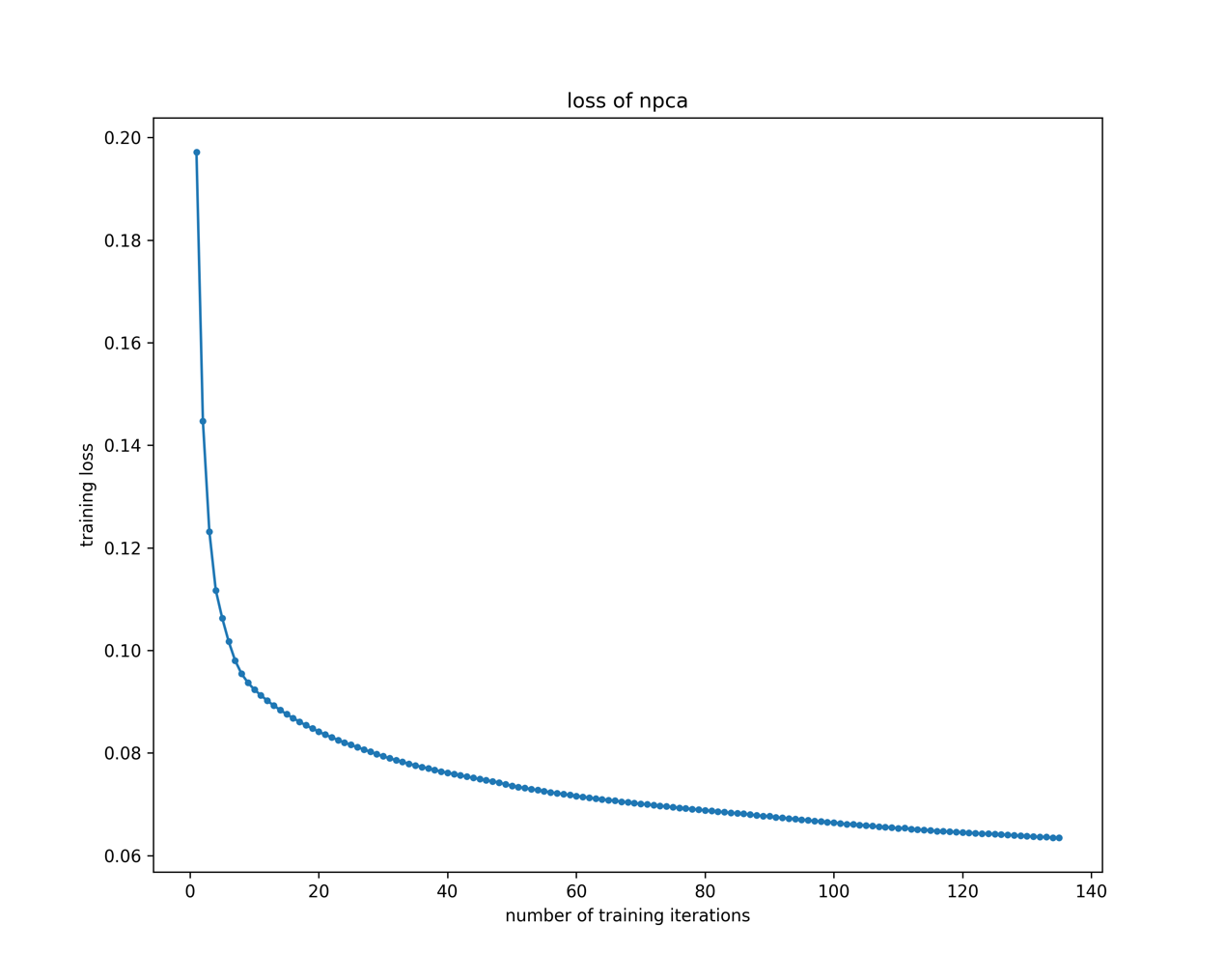


**Figure S30.** the loss of nPCA of housing dataset


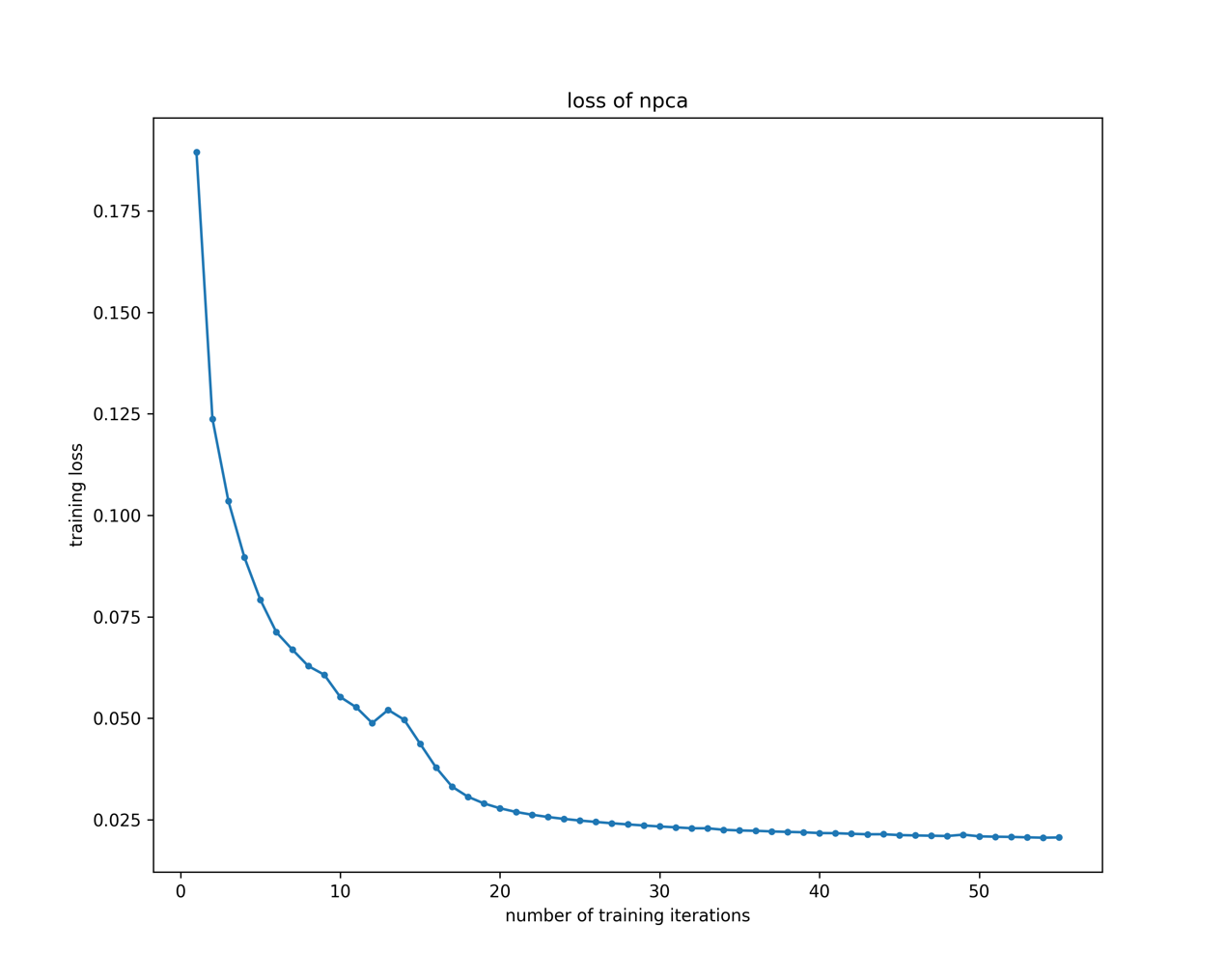


**Figure S31.** the loss of nPCA of indian liver patient dataset


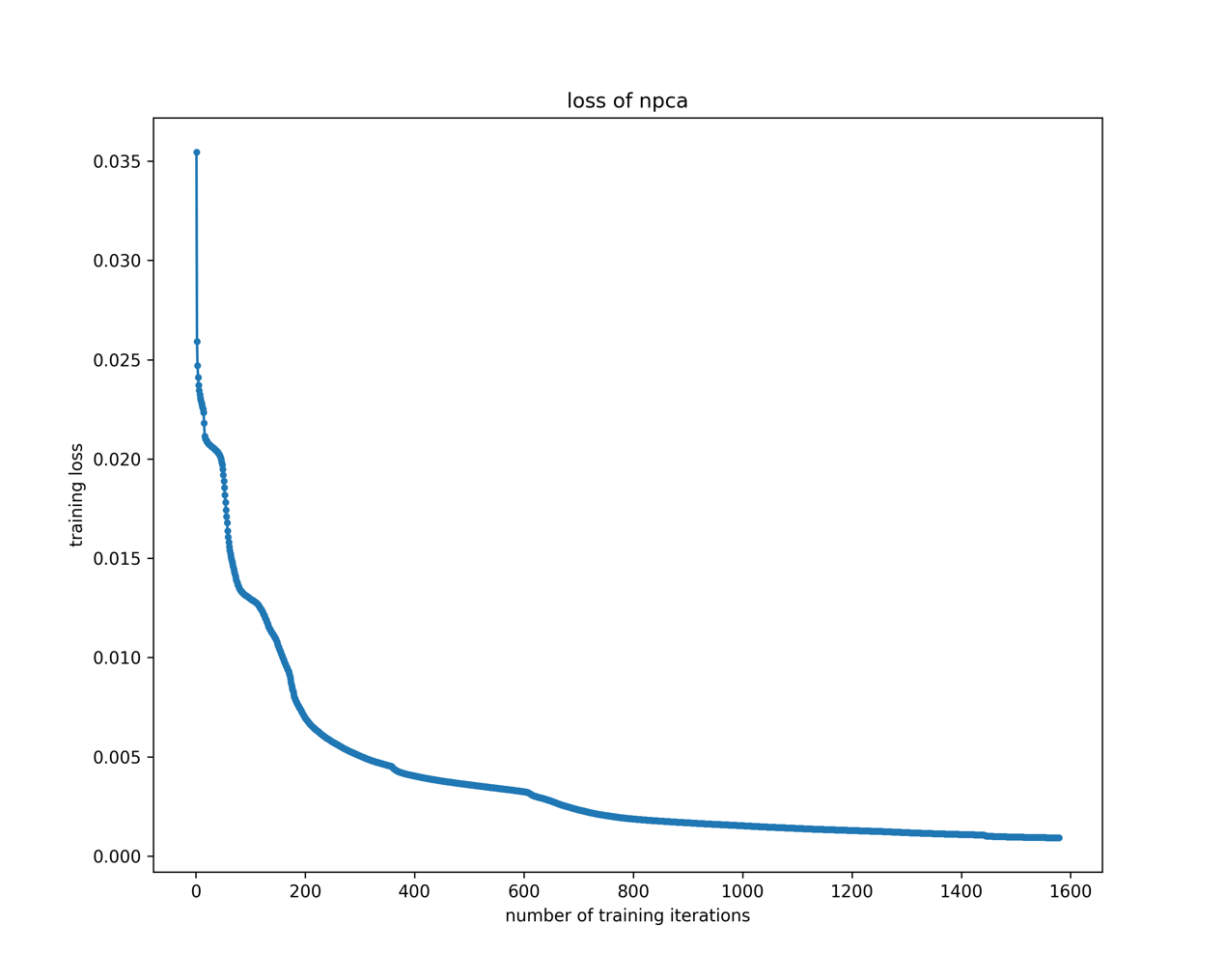


**Figure S32.** the loss of nPCA of iris dataset


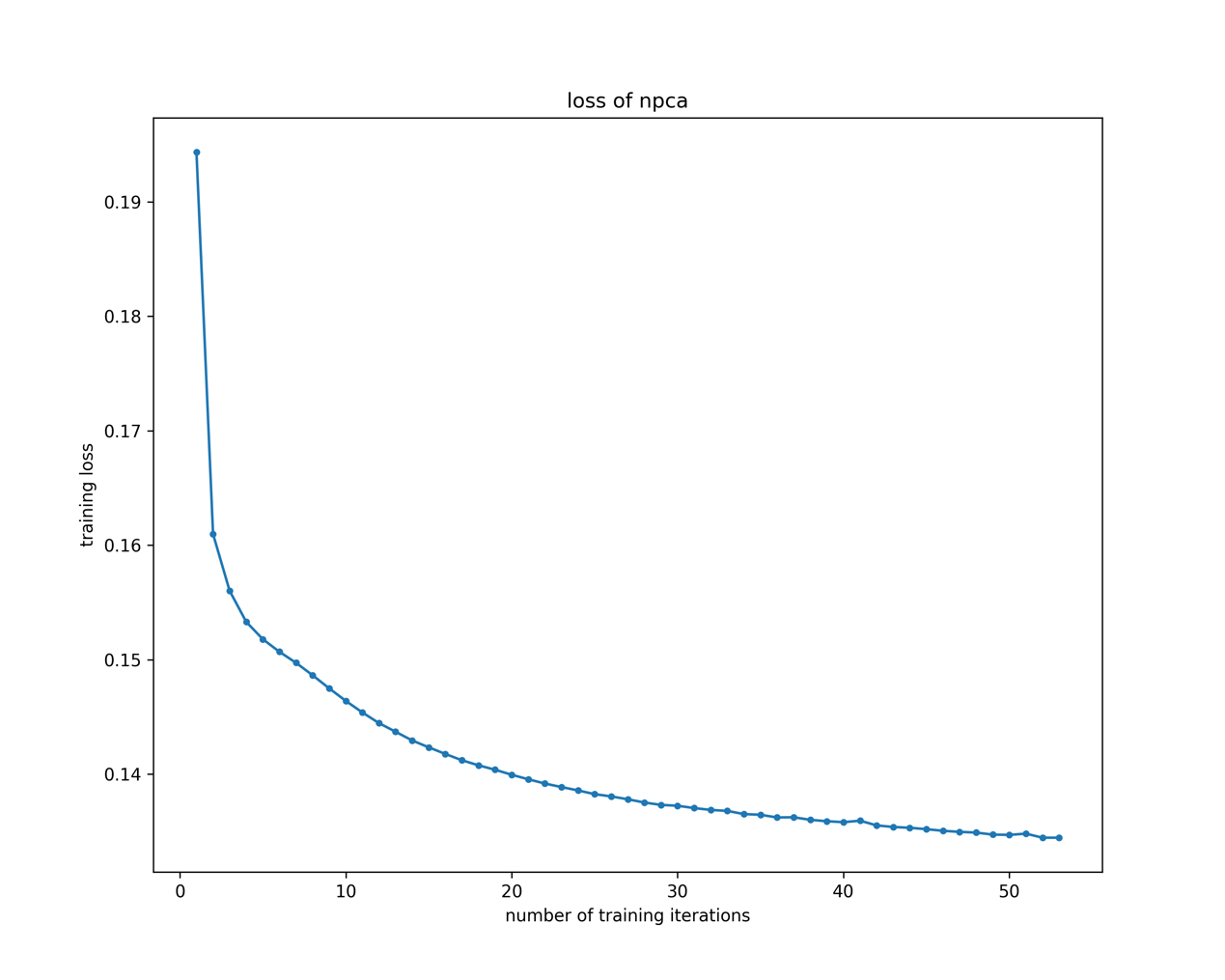


**Figure S33.** the loss of nPCA of parkinsons telemonitoring dataset


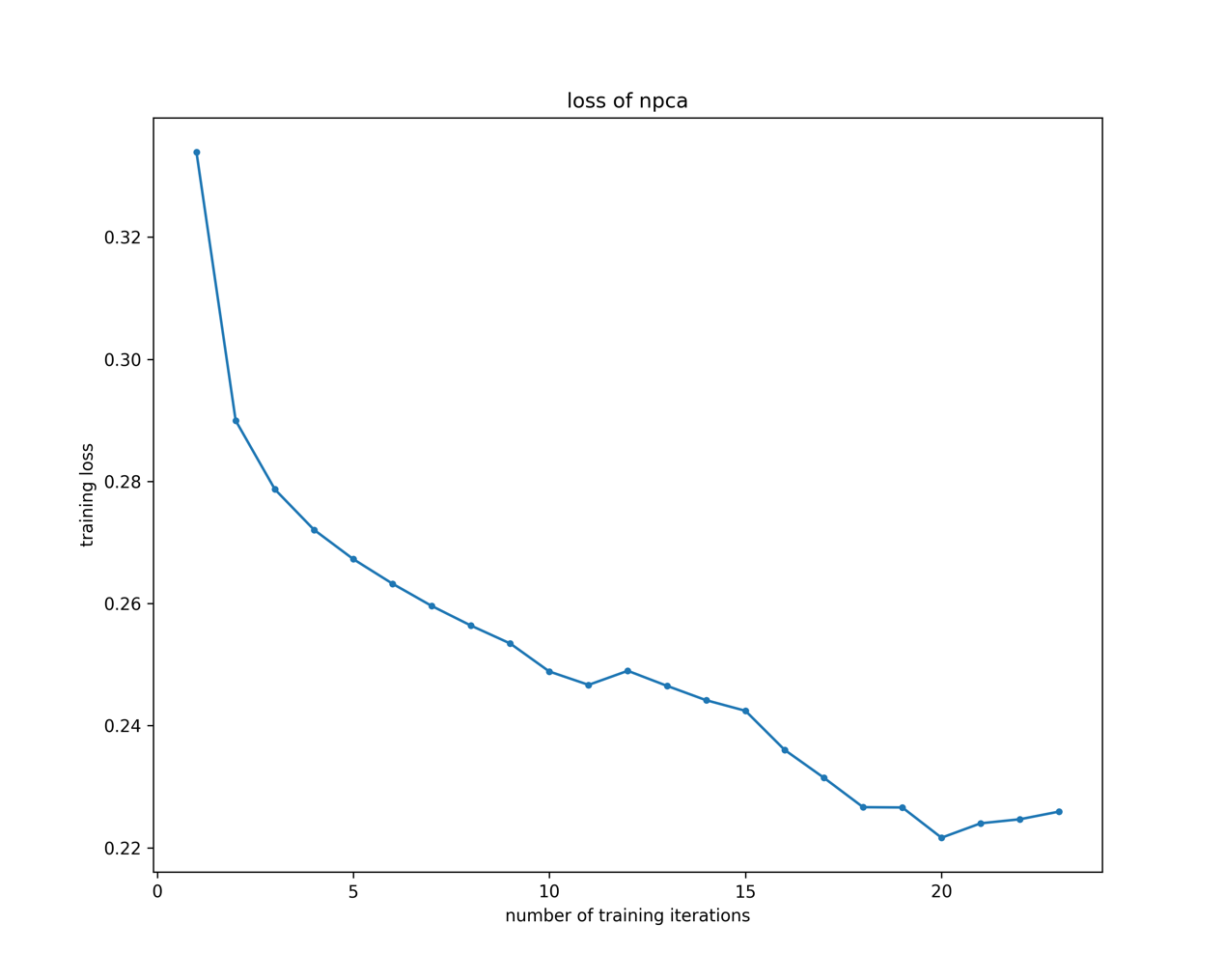


**Figure S34.** the loss of nPCA of QSAR biodegradation dataset


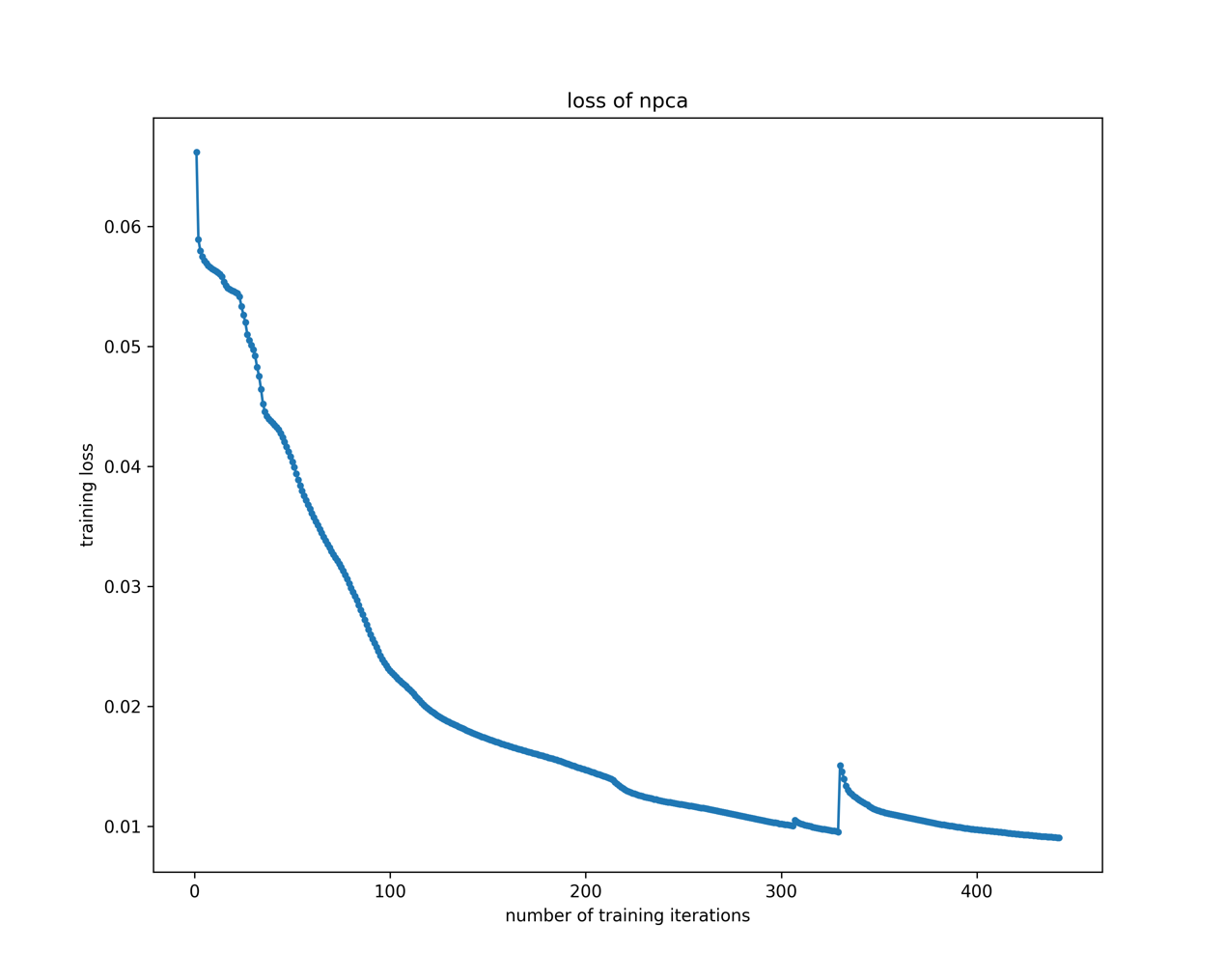


**Figure S35.** the loss of nPCA of seeds dataset


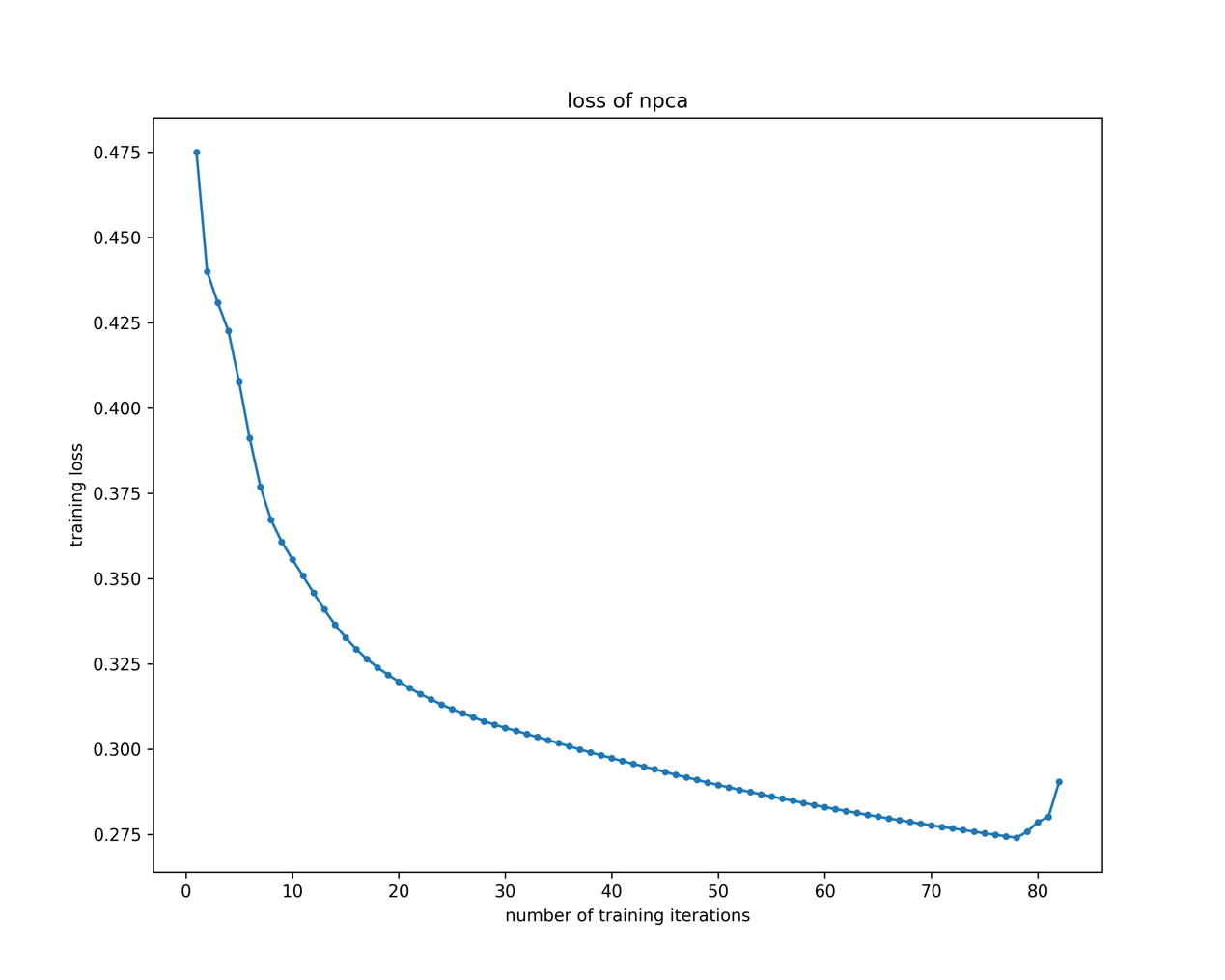


**Figure S36.** the loss of nPCA of Baron human1 of 200 highly variable genes dataset


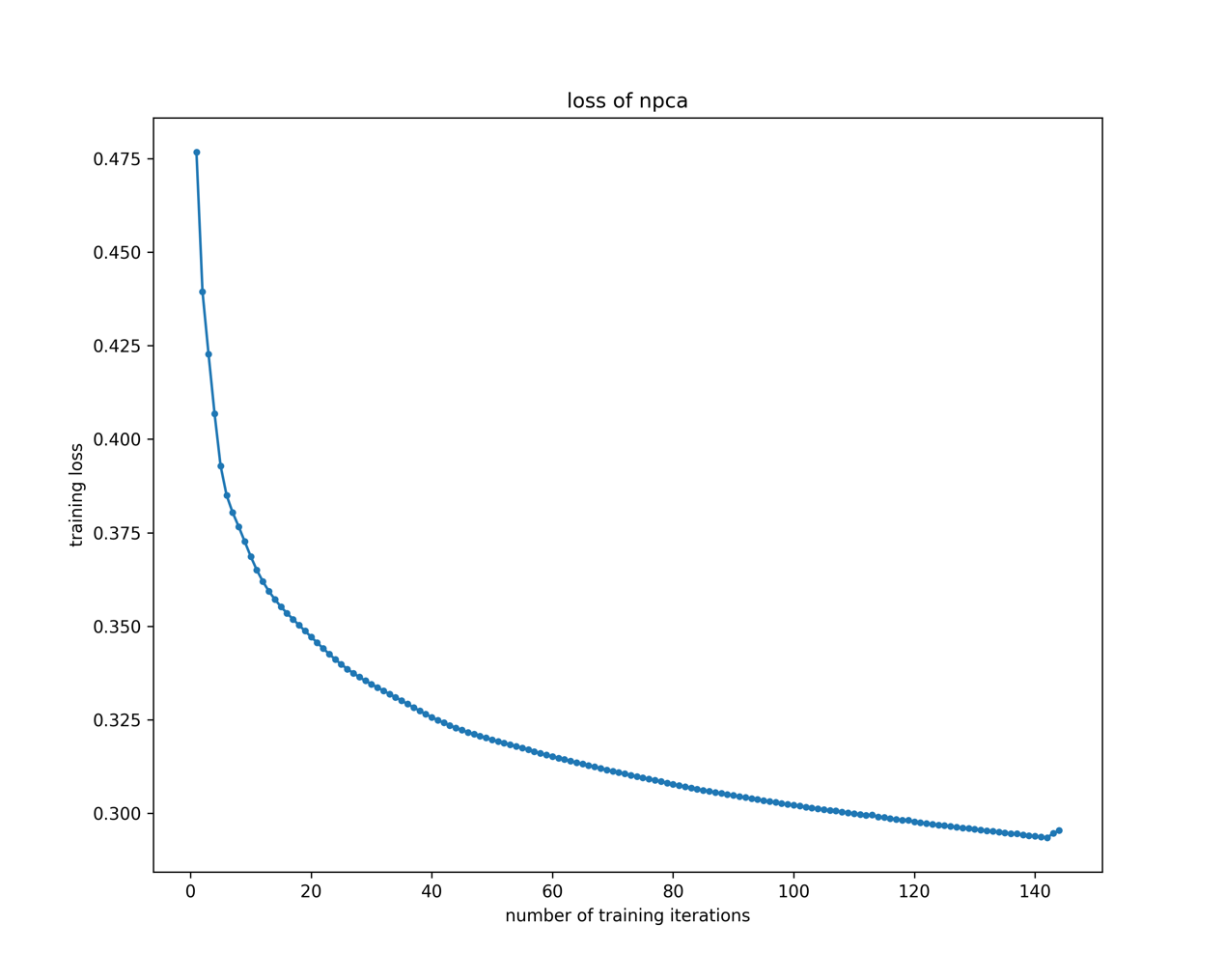


**Figure S37.** the loss of nPCA of Baron human1 of 500 highly variable genes dataset


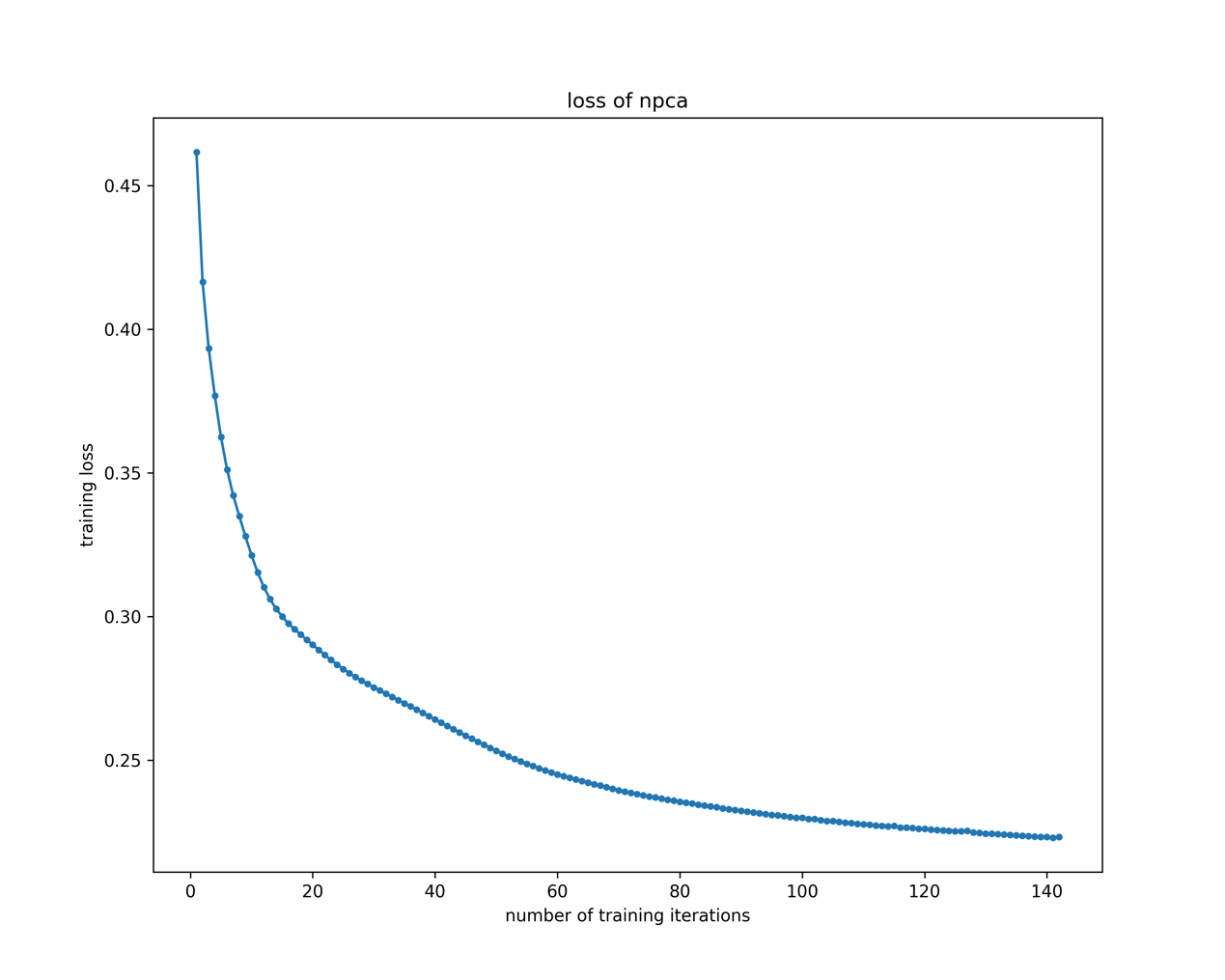


**Figure S38.** the loss of nPCA of Baron human2 of 200 highly variable genes dataset


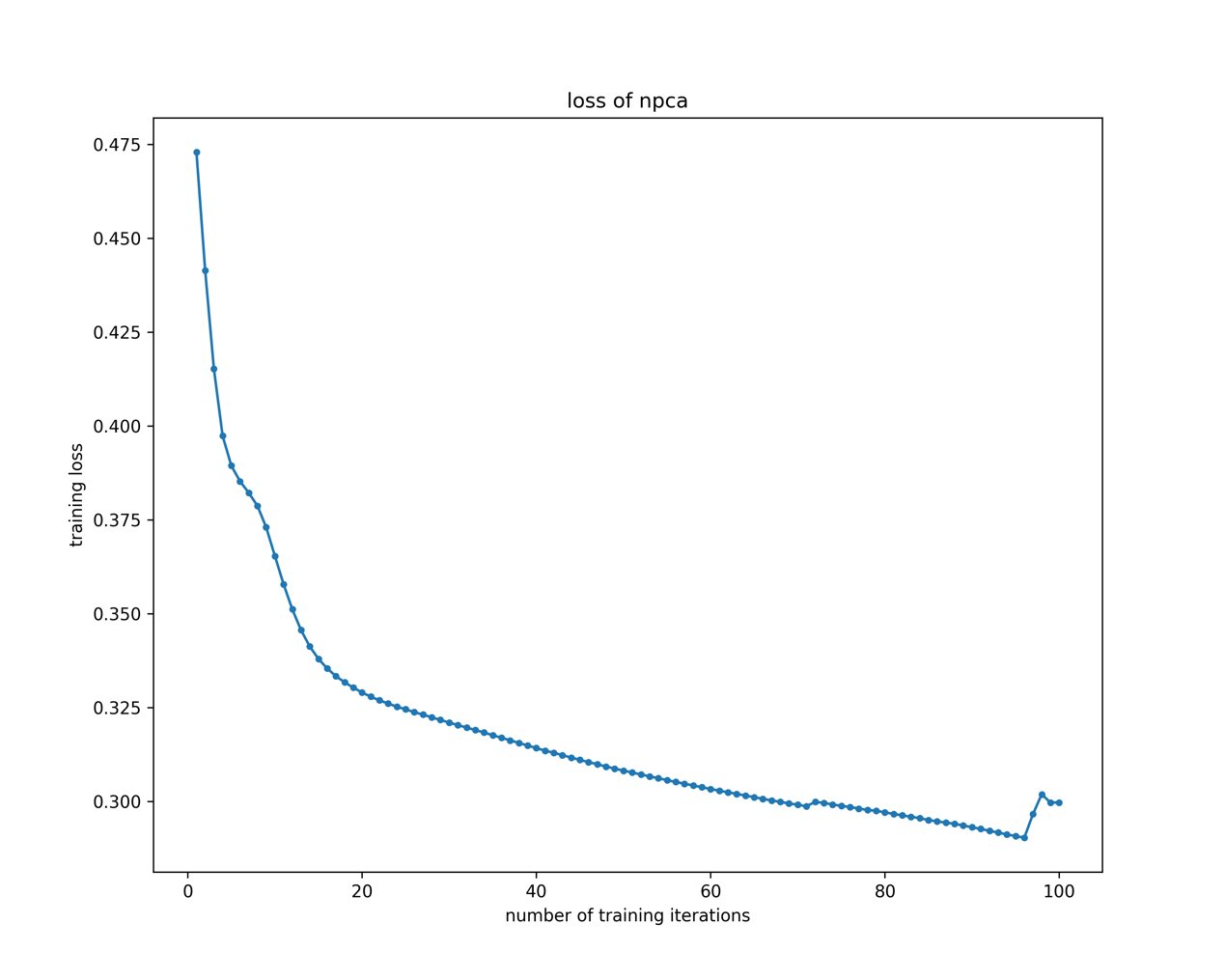


**Figure S39.** the loss of nPCA of Baron human2 of 500 highly variable genes dataset


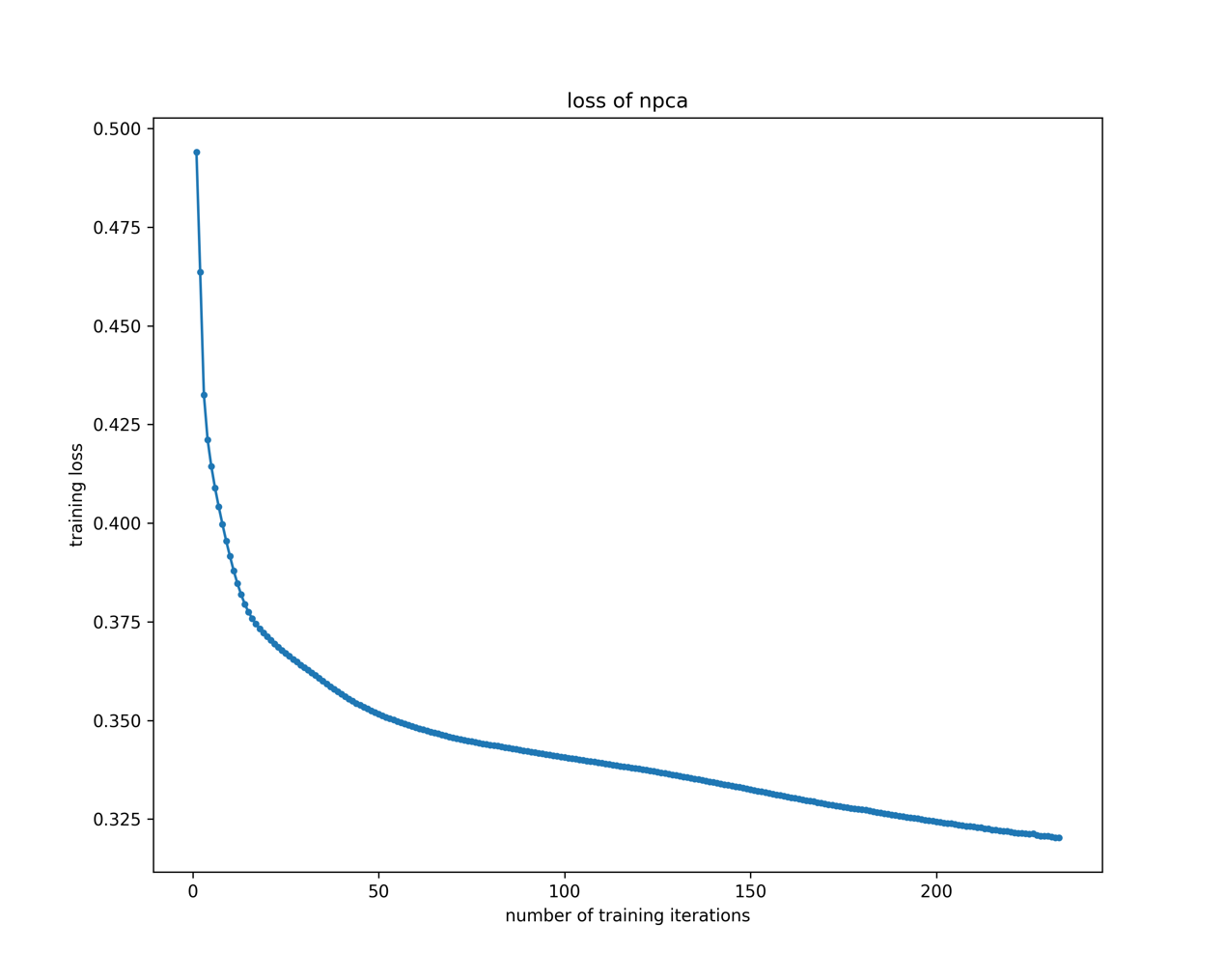


**Figure S40.** the loss of nPCA of Baron human2 of 1000 highly variable genes dataset


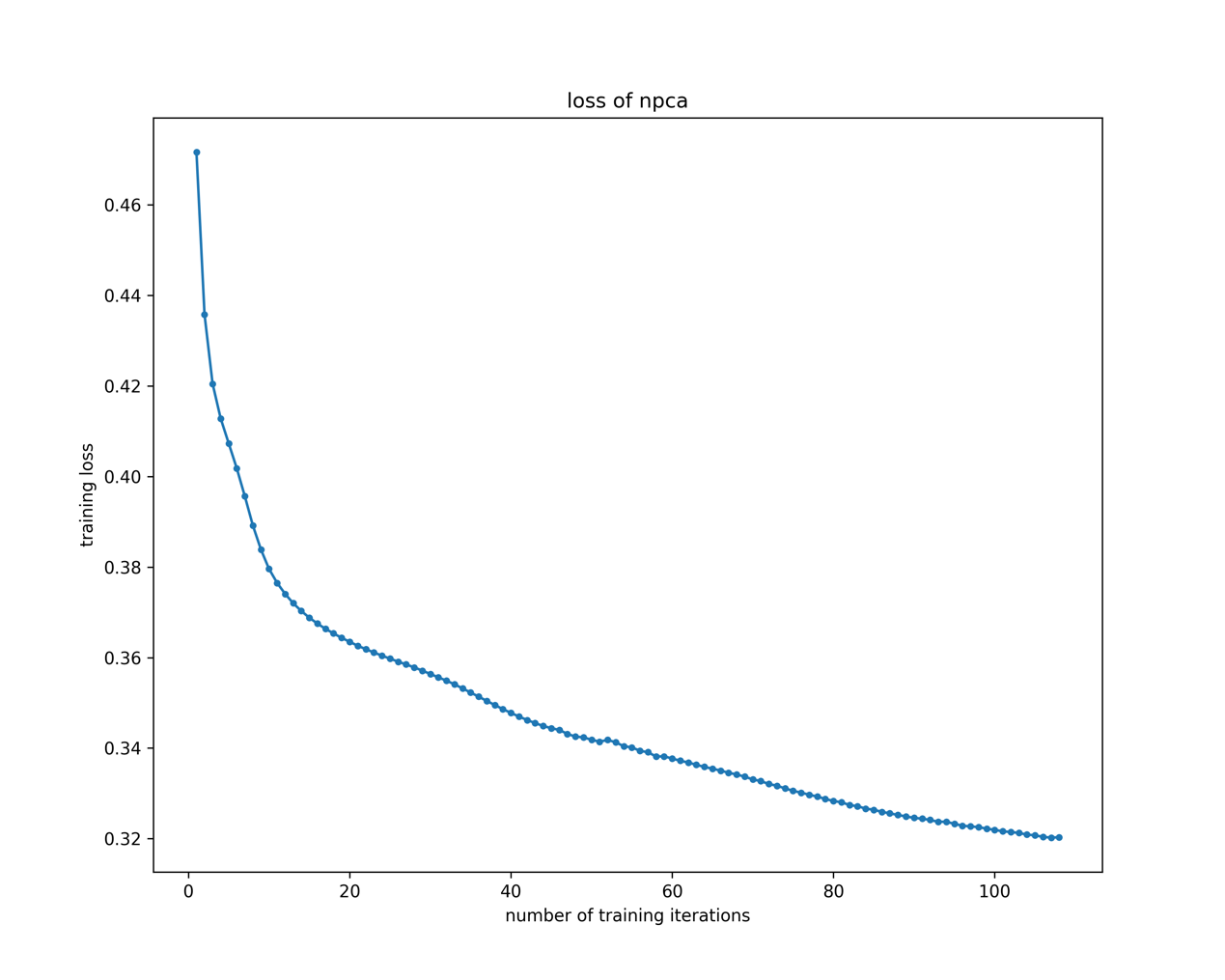


**Figure S41.** the loss of nPCA of Baron human3 of 200 highly variable genes dataset


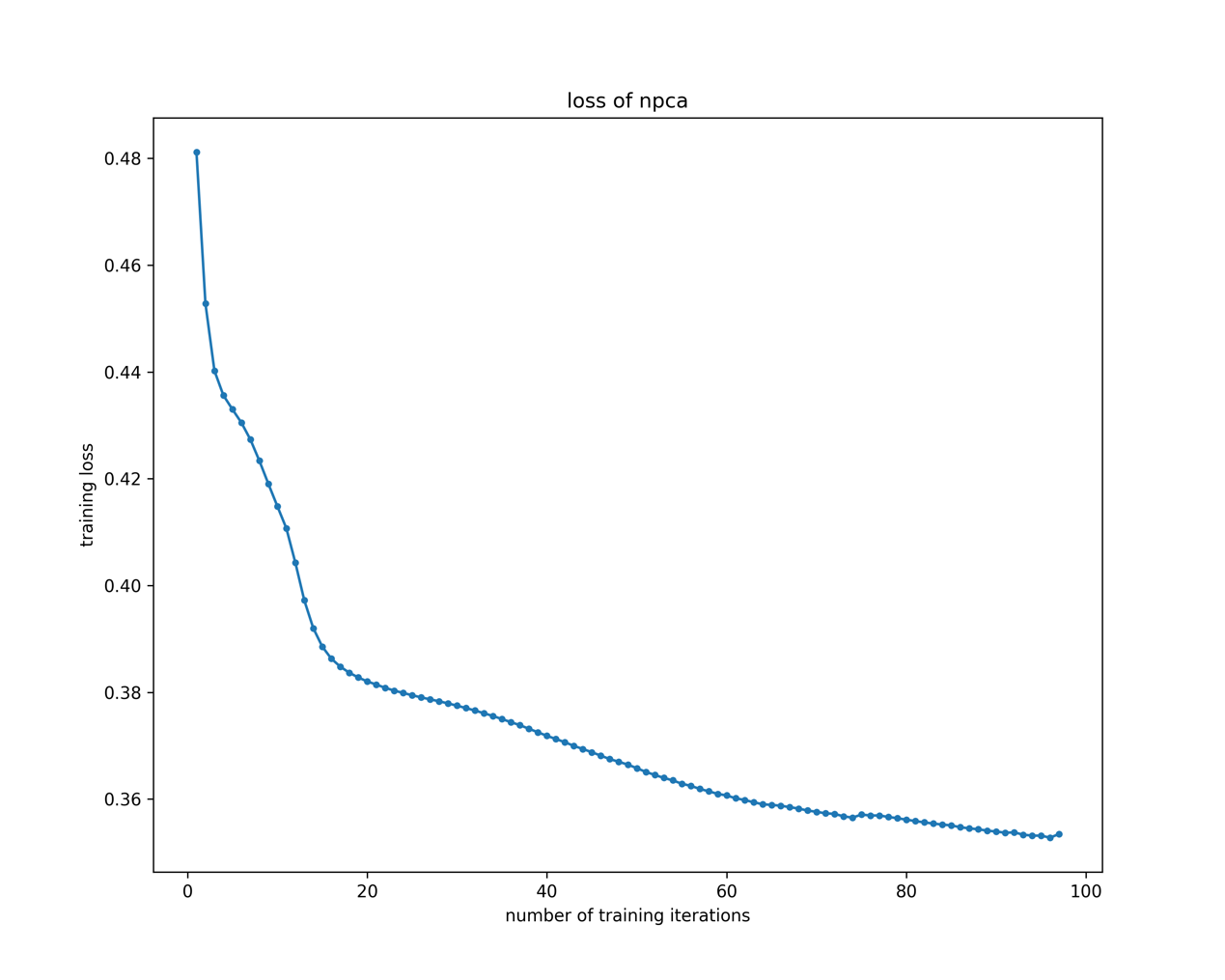


**Figure S42.** the loss of nPCA of Baron human3 of 500 highly variable genes dataset


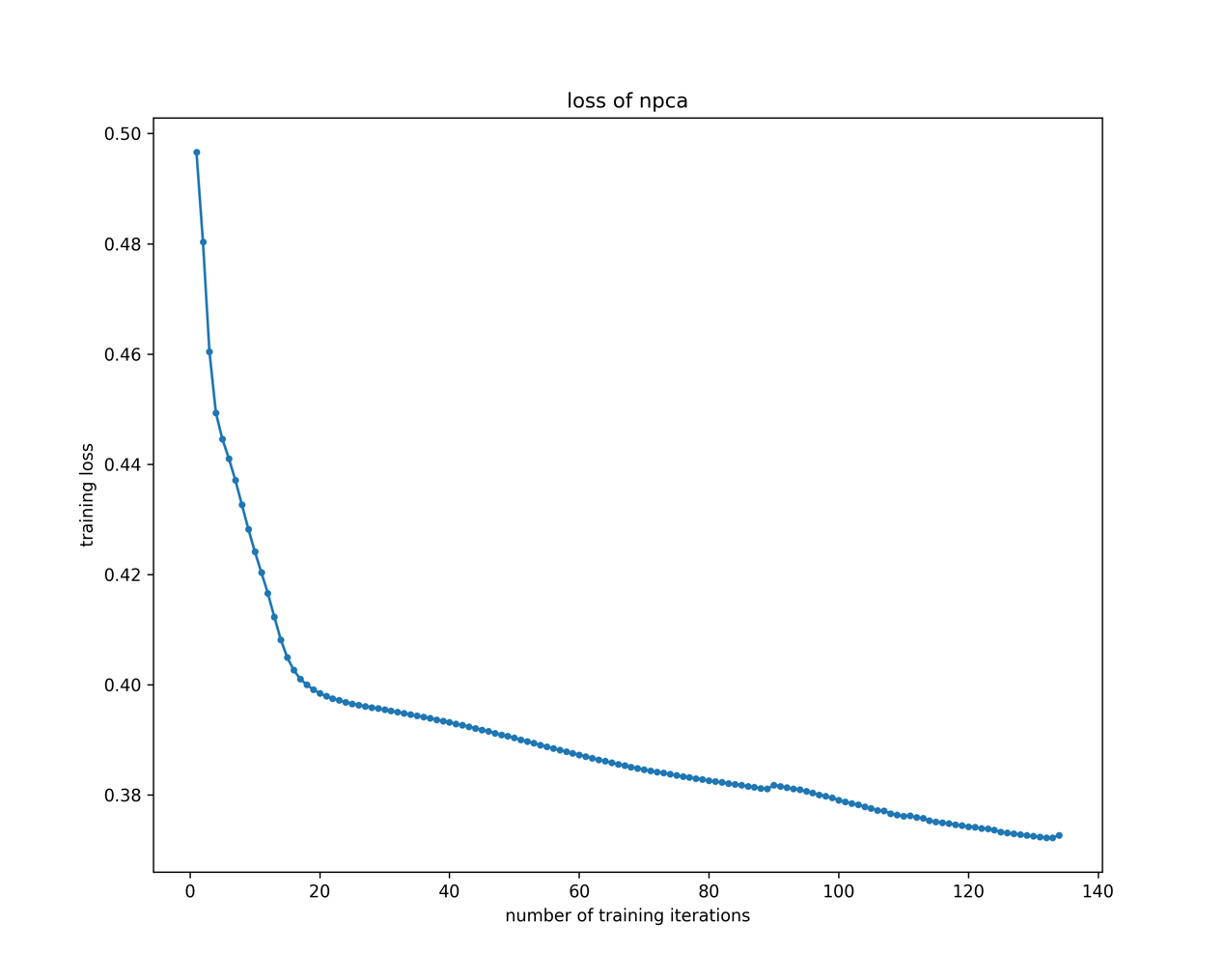


**Figure S43.** the loss of nPCA of Baron human3 of 1000 highly variable genes dataset


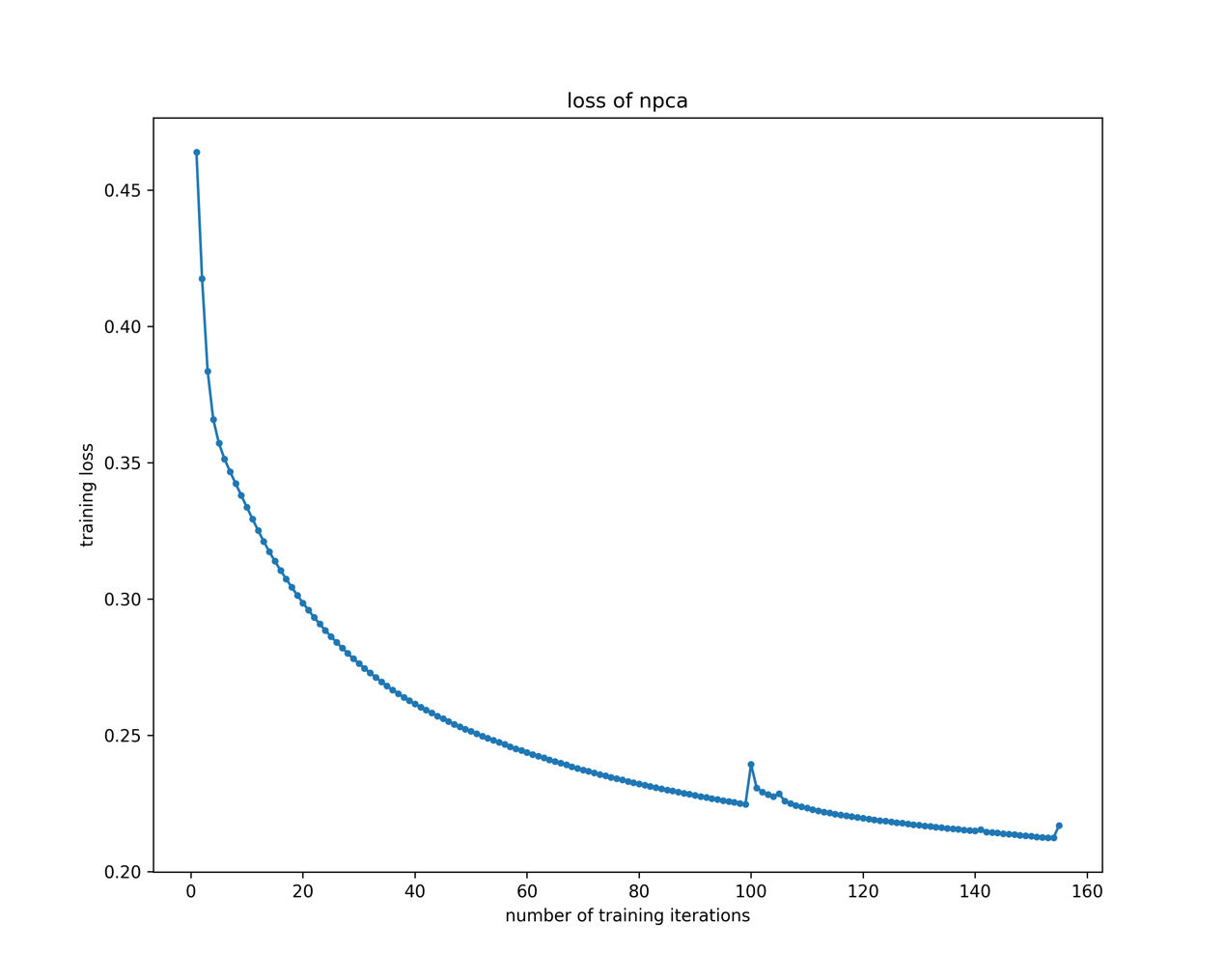


**Figure S44.** the loss of nPCA of Baron human4 of 200 highly variable genes dataset


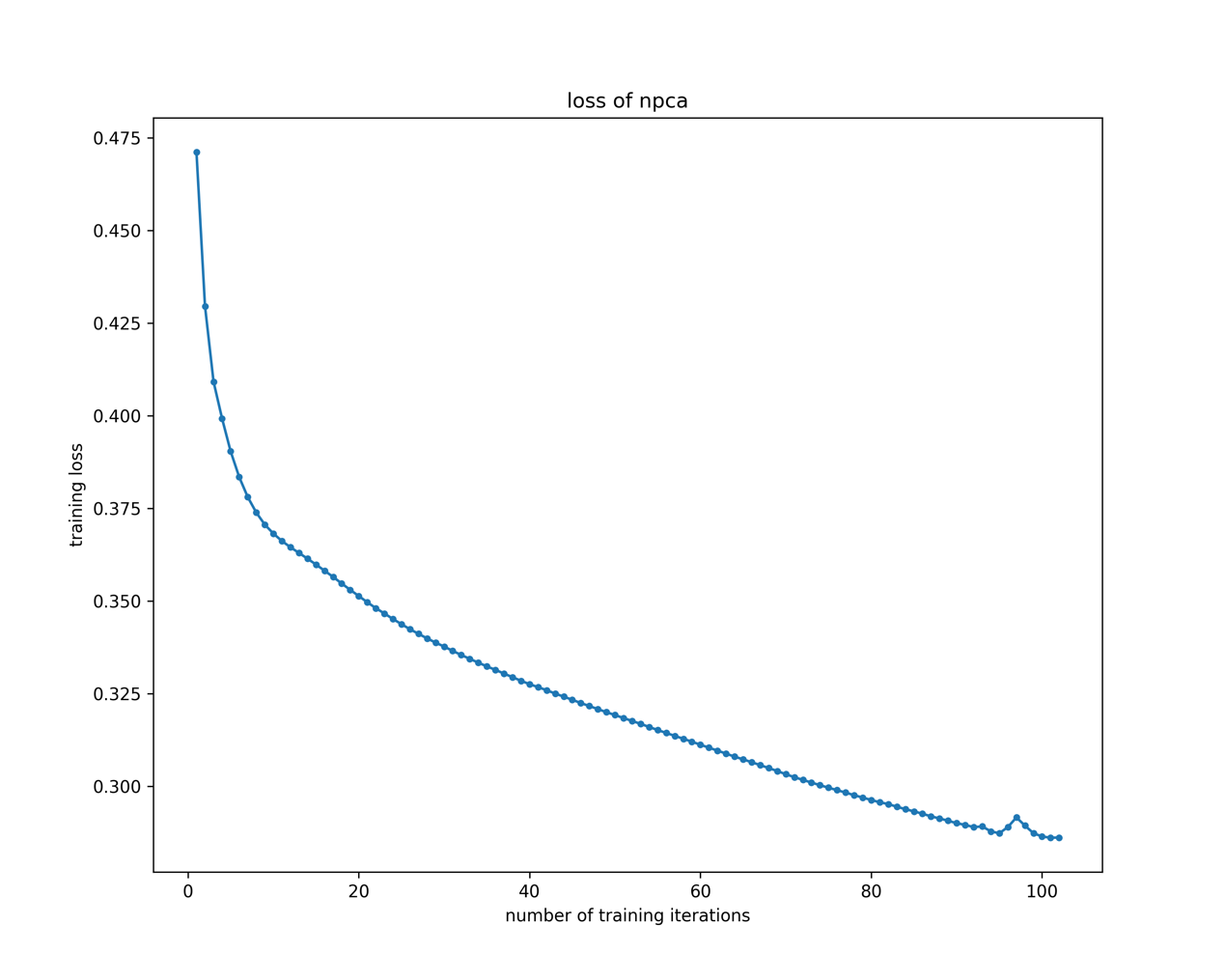


**Figure S45.** the loss of nPCA of Baron human4 of 500 highly variable genes dataset


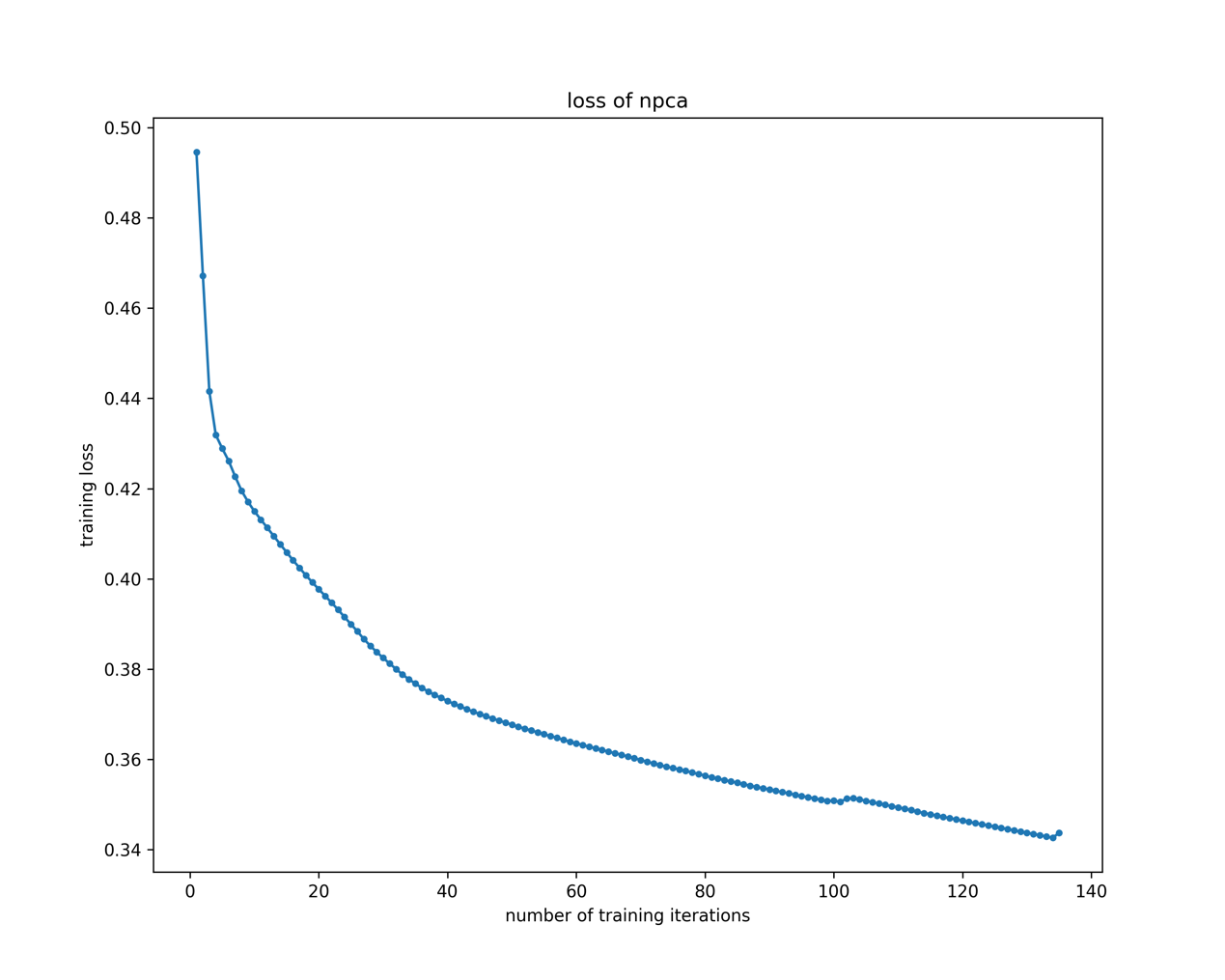


**Figure S46.** the loss of nPCA of Baron human4 of 1000 highly variable genes dataset


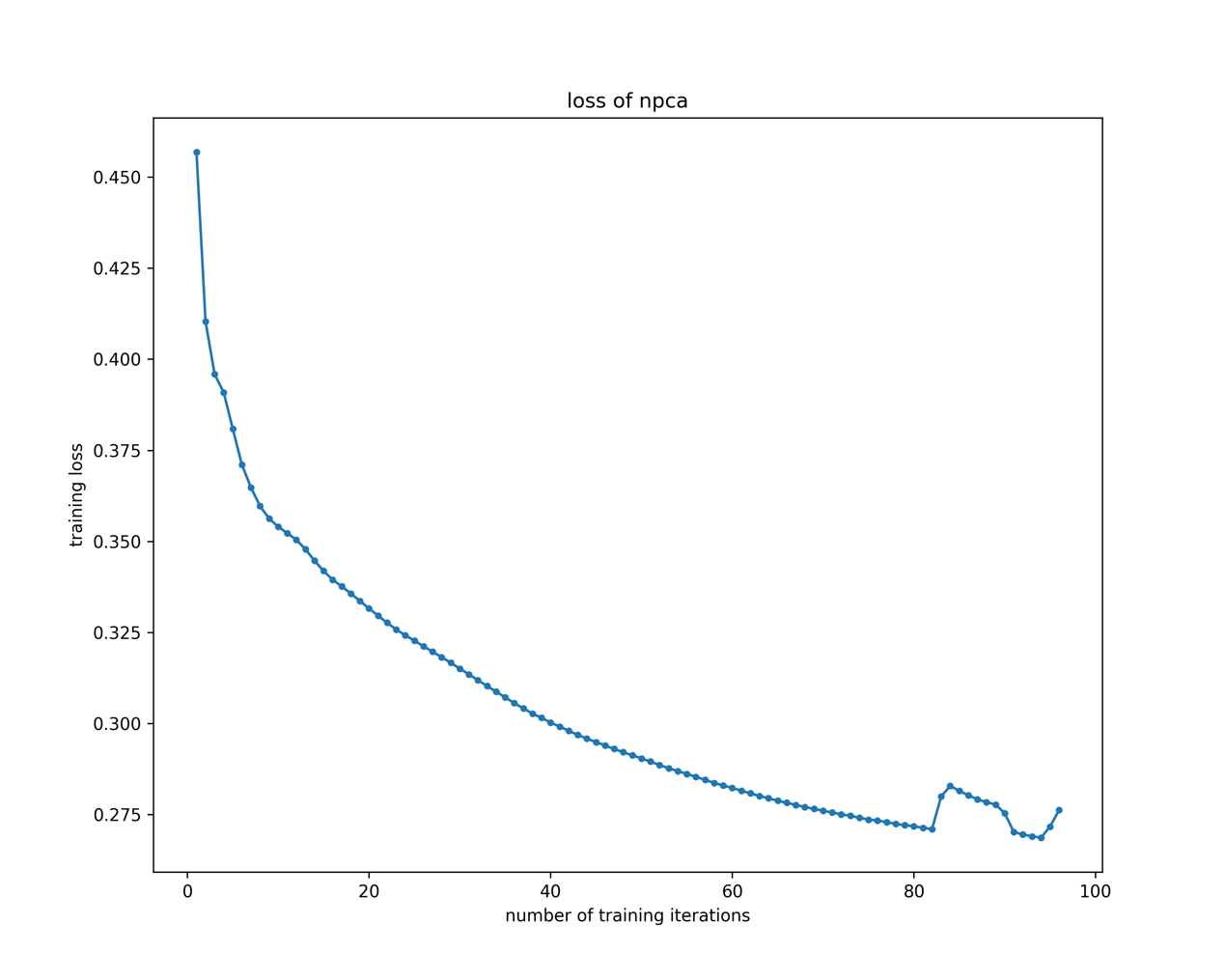


**Figure S47.** the loss of nPCA of Baron mouse1 of 200 highly variable genes dataset


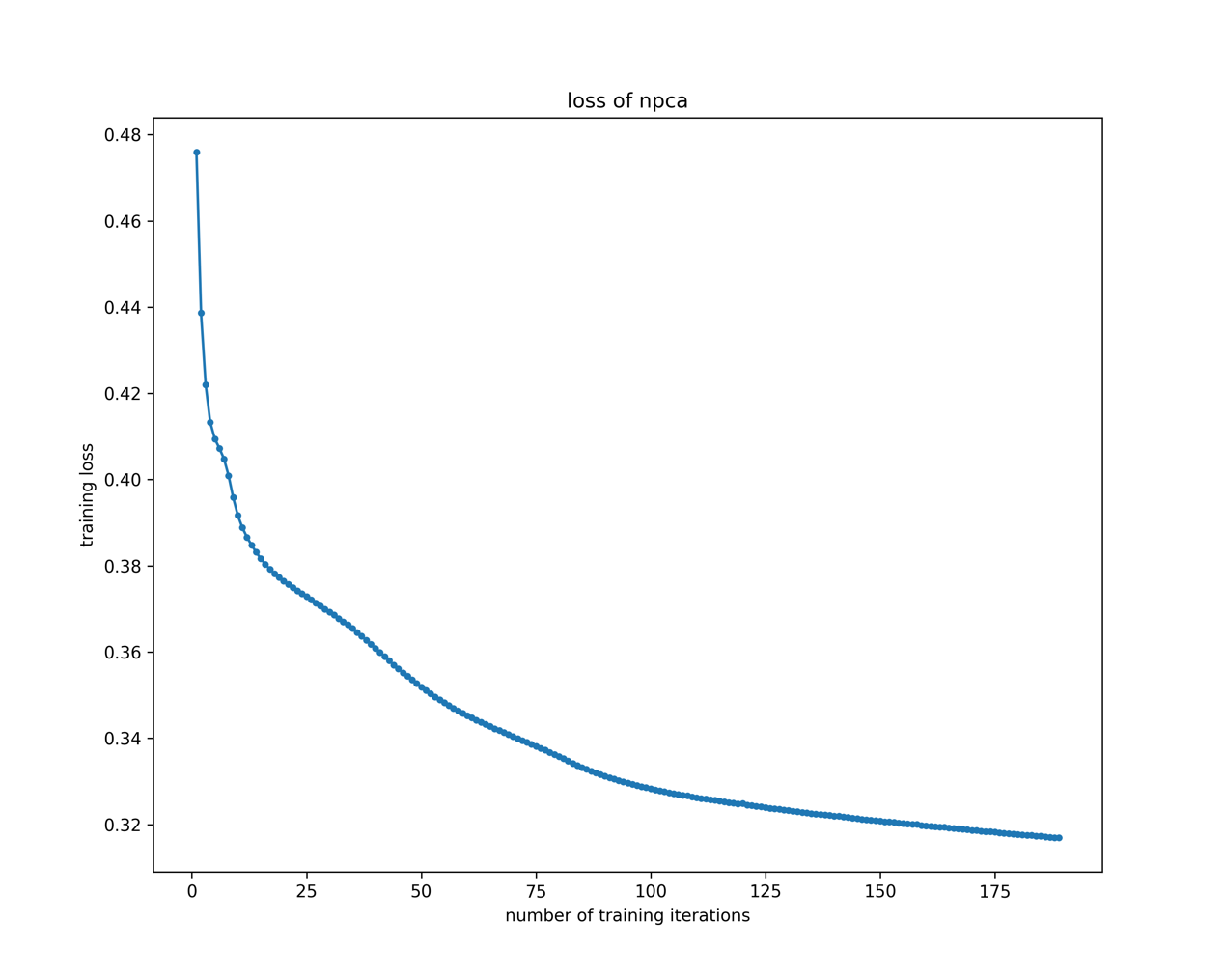


**Figure S48.** the loss of nPCA of Baron mouse1 of 500 highly variable genes dataset


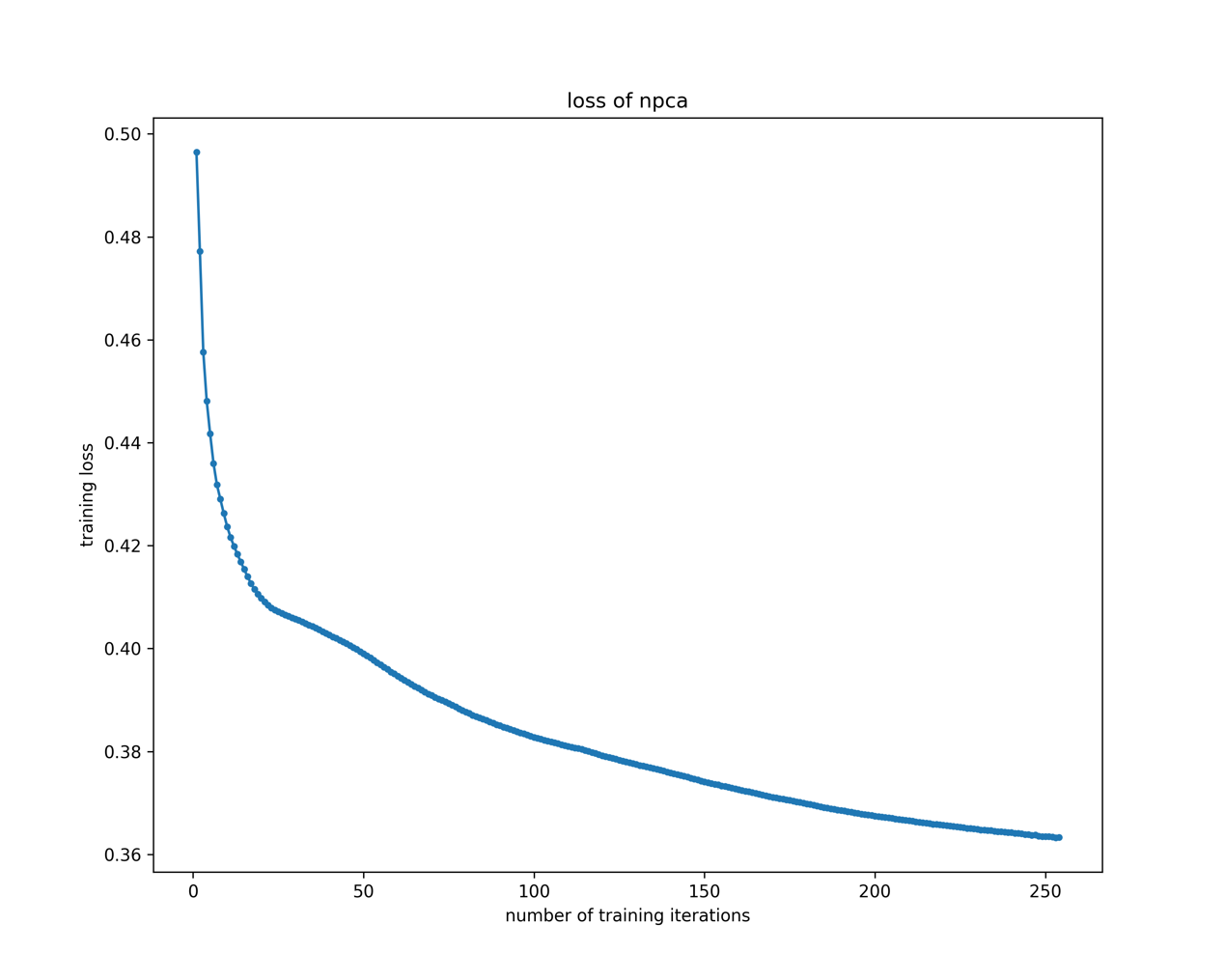


**Figure S49.** the loss of nPCA of Baron mouse1 of 1000 highly variable genes dataset


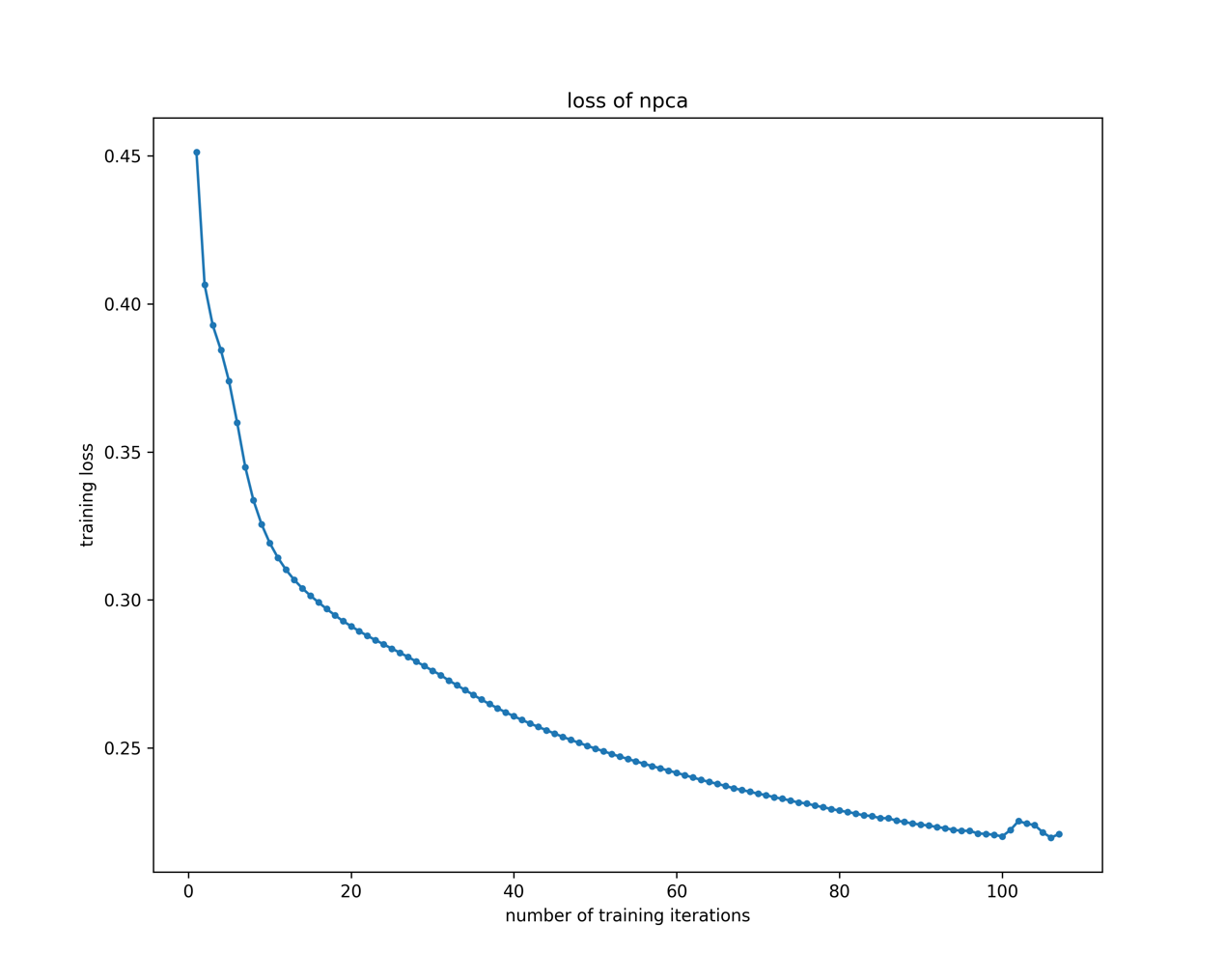


**Figure S50.** the loss of nPCA of Baron mouse2 of 200 highly variable genes dataset


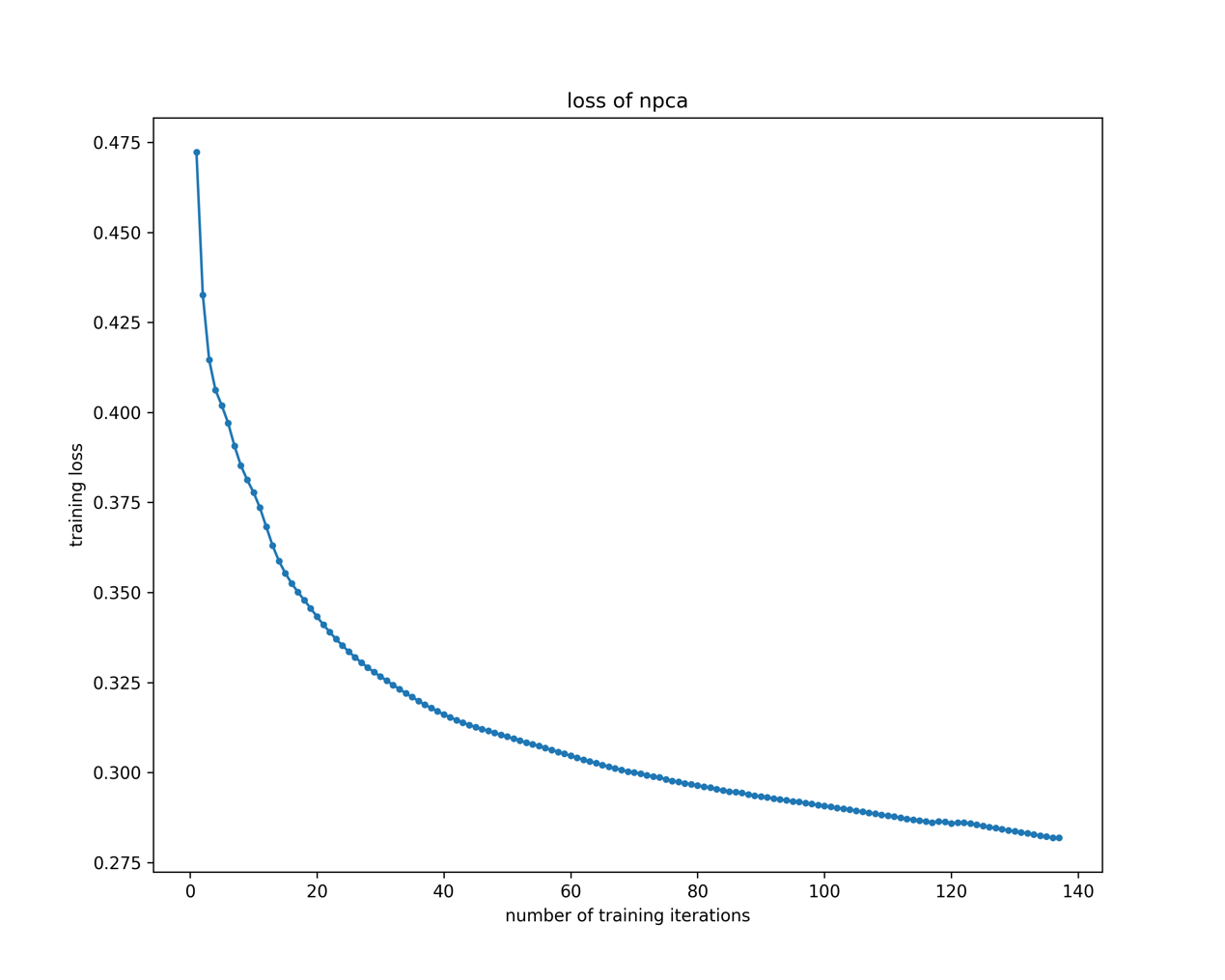


**Figure S51.** the loss of nPCA of Baron mouse2 of 500 highly variable genes dataset


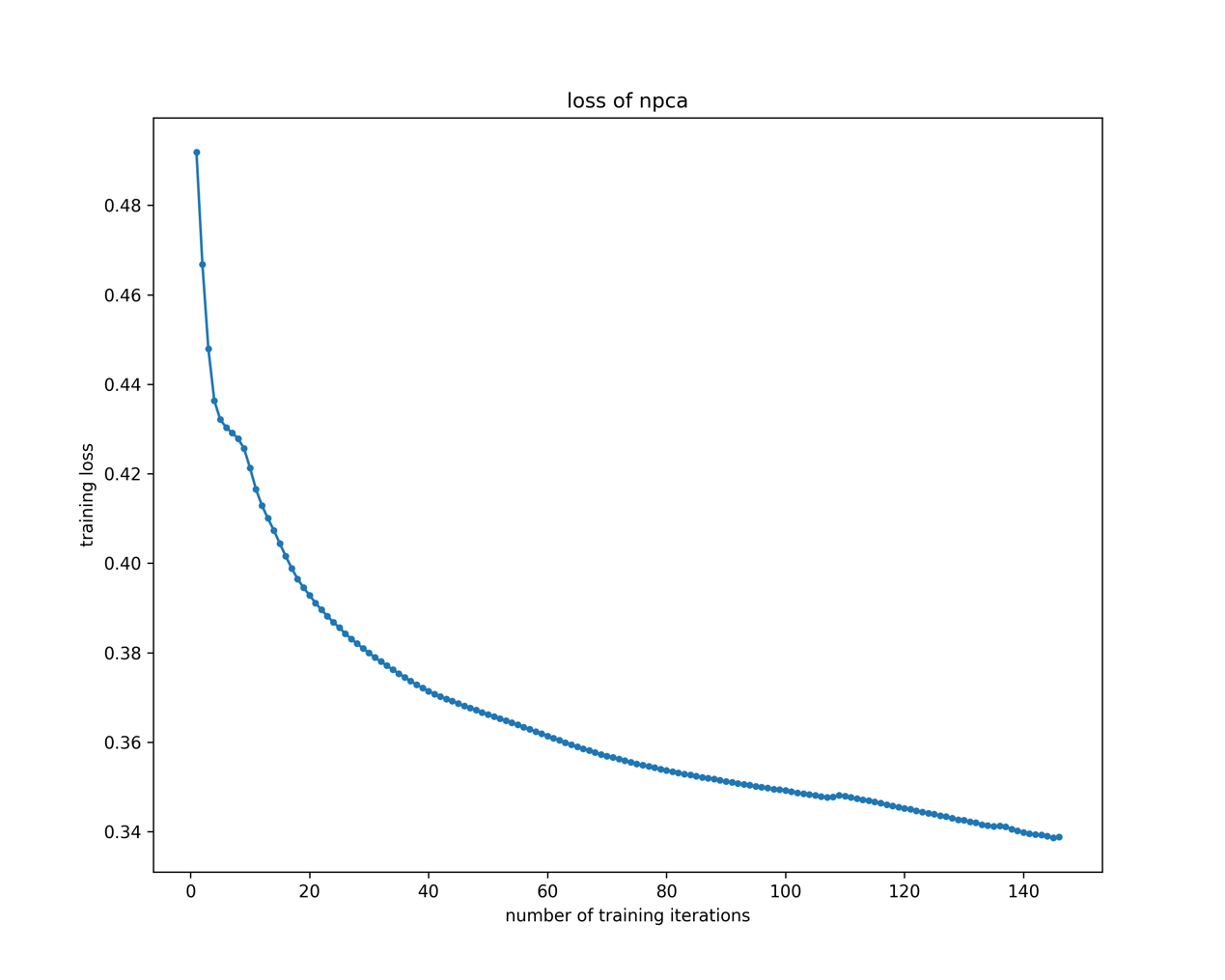


**Figure S52.** the loss of nPCA of Baron mouse2 of 1000 highly variable genes dataset
